# Recent Contributions of Proteomics to Our Understanding of Reversible N^ε^-Lysine Acylation in Bacteria

**DOI:** 10.1021/acs.jproteome.3c00912

**Published:** 2024-03-05

**Authors:** Liya Popova, Rachel A. Carr, Valerie J. Carabetta

**Affiliations:** Department of Biomedical Sciences, Cooper Medical School of Rowan University, Camden, New Jersey 08103, United States

**Keywords:** acetyl, acetylome, KDAC, KAT, acetylation, succinylation, succinylome, post-translational modification, PTM

## Abstract

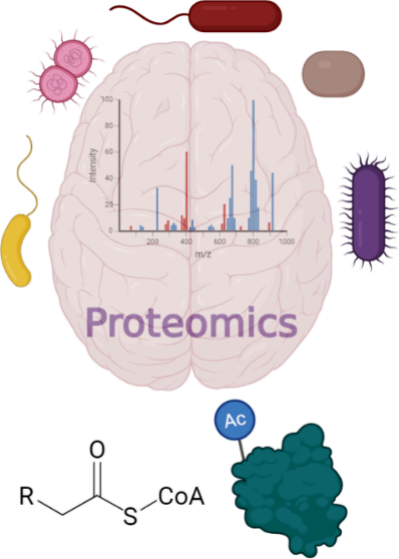

Post-translational modifications (PTMs) have been extensively studied in both eukaryotes and prokaryotes. Lysine acetylation, originally thought to be a rare occurrence in bacteria, is now recognized as a prevalent and important PTM in more than 50 species. This expansion in interest in bacterial PTMs became possible with the advancement of mass spectrometry technology and improved reagents such as acyl-modification specific antibodies. In this Review, we discuss how mass spectrometry-based proteomic studies of lysine acetylation and other acyl modifications have contributed to our understanding of bacterial physiology, focusing on recently published studies from 2018 to 2023. We begin with a discussion of approaches used to study bacterial PTMs. Next, we discuss newly characterized acylomes, including acetylomes, succinylomes, and malonylomes, in different bacterial species. In addition, we examine proteomic contributions to our understanding of bacterial virulence and biofilm formation. Finally, we discuss the contributions of mass spectrometry to our understanding of the mechanisms of acetylation, both enzymatic and nonenzymatic. We end with a discussion of the current state of the field and possible future research avenues to explore.

## Introduction

Numerous post-translational modifications (PTMs) have been recognized in both eukaryotes and prokaryotes including phosphorylation, acetylation, and methylation. While the exact number of PTMs in bacteria is challenging to estimate, it is known that over 400 distinct types of PTMs have been identified in eukaryotes and research in this field is continually expanding.^1^ PTMs were first identified among eukaryotic proteins in the 20th century, with the discovery of phosphorylated serine residues in vitellin in 1906^2^ and protein acetylation and methylation on eukaryotic histones in the 1960s.^3^ For a long time, acetylation and other acyl modifications were thought to be nonexistent in bacteria. However, in the late 1990s, the chemotaxis protein CheY was discovered to undergo acetylation, representing the first documented example of bacterial protein acetylation.^4,5^  Protein acetylation was not truly recognized as a relevant PTM in bacteria until a decade after this initial report, with the first characterization of the *Escherichia coli* acetylome using mass spectrometry-based (MS) proteomics.^6^ Since this initial report in 2008, the field of bacterial lysine acetylation has exploded. Now, N^ε^-lysine acetylation is recognized as a common and widespread PTM that affects hundreds of proteins in both eukaryotes and prokaryotes. The identification of PTMs in bacteria has significantly expanded with the advancement of reagents and MS instrumentation, allowing for the exploration of the bacterial PTM landscape more comprehensively than ever before. From these studies, an understanding emerged that PTMs play crucial roles in controlling protein function, stability, interactions, and subcellular localization, with important implications in essential pathways and virulence. The work done in this field from 2017 and earlier has been extensively reviewed.^7−14^ Here, we describe how MS-based proteomic studies of lysine acylation have contributed to our understanding of bacterial physiology, focusing on studies published from 2018 to 2023. We begin with a discussion of MS approaches used to study PTMs, including quantitative approaches. Next, we discuss new updates in our understanding of lysine acetylation and other acyl modifications such as succinylation, butyrylation, and propionylation. In addition, we examine advancements in our understanding of bacterial virulence, including biofilm formation and the mechanism of acetylation. We end with a discussion of the current state of the field and possible future research avenues to explore. MS-based proteomics has become a cornerstone in modern biological and biomedical research, contributing to our fundamental understanding of complex biological systems.^15^

## MS Approaches to Study PTMs

The necessity of bacterial cells to adapt to the changing, often unfavorable, environment can be achieved by several intracellular mechanisms, such as transcription, translation, and formation of proteoforms. The latter plays a significant role in the modification of the cellular physiological state and metabolism, allowing bacteria to quickly adjust intracellular processes without the necessity of the energy-intensive and resource-consuming processes of transcription and translation. The proteoforms, which include forms of the protein modified by covalent PTMs, can vary greatly in their functional and biological roles. MS-based proteomics opened the door to analyze the entire, endogenous proteome in cells or organisms in a short amount of time that is relatively cost-effective. This is especially relevant when compared to alternative proteomic approaches that require the production of large amounts of recombinant, tagged proteins or the production of specific antibodies. These techniques cannot match the throughput, sensitivity, and speed of MS-based proteomics.

**Figure 1:**
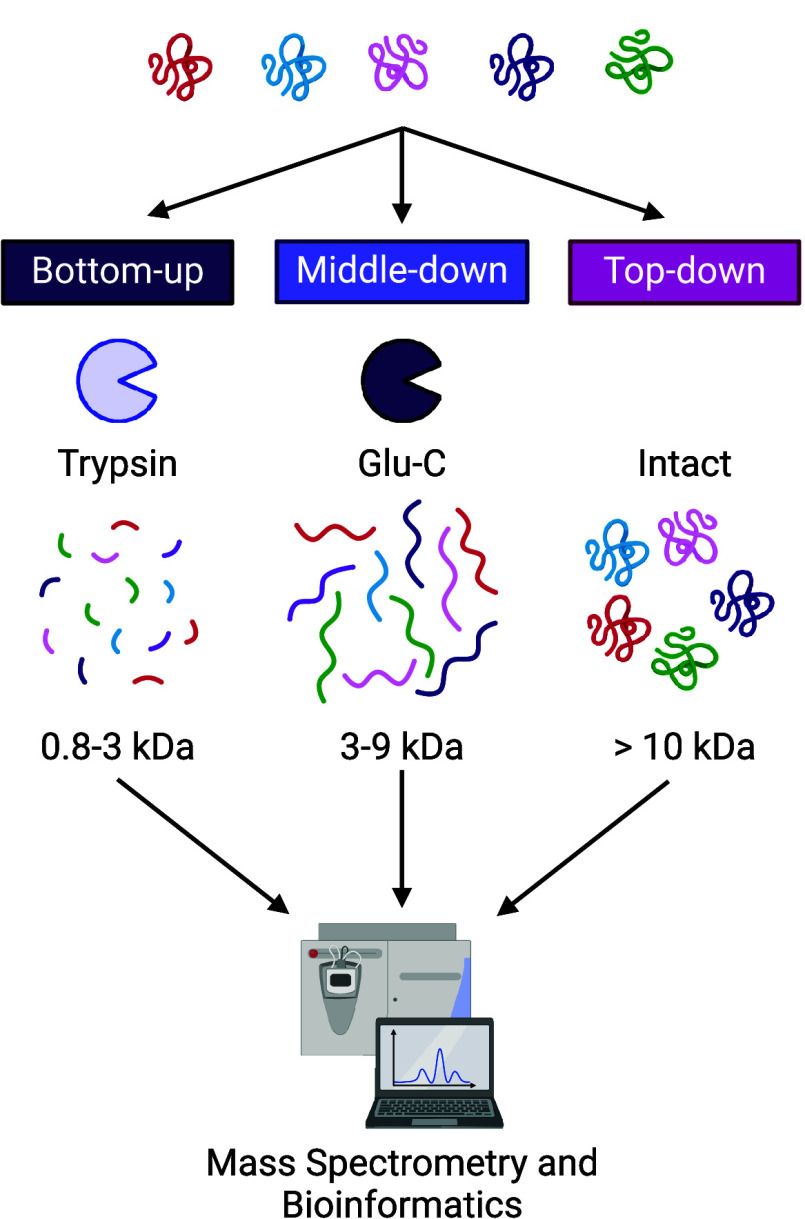
Bottom-up, middle-down, and top-down MS strategies. For the bottom-up approach, proteins are digested into small peptides (0.8–3 kDa), most often using the enzyme trypsin. Each peptide will have an arginine or lysine at the C-terminus. For middle-down analysis, proteins are partially digested using Glu-C or Asp-N, which yields longer peptides (3–9 kDa). Glu-C peptides will have a glutamate at the C-terminus. The top-down approach does not use digestion and analyzes intact proteins. No matter which approach is used, the peptides or proteins are analyzed by MS and bioinformatics, depending on the specific conditions required for each approach.

MS analysis of proteomics can be broadly divided into bottom-up (“shotgun”), middle-down, and top-down techniques (Figure 1). Bottom-up involves enzymatic digestion of proteins into small fragments; middle-down involves a limited digestion creating larger fragments, while top-down directly detects proteins without digestion. The bottom-up approach has several pros and cons for the analysis of PTMs.^16,17^ The gold-standard for enzymatic digestion for the bottom-up approach is trypsin, which cleaves C-terminally to lysine and arginine residues, but others can be used successfully. One advantage of this approach is that it is the most sensitive of the strategies. The use of peptides over proteins is advantageous because they are more easily separated by reverse-phase liquid chromatography, ionize well, and produce predictable fragmentation patterns, which facilitate downstream analyses. Finally, it may simplify or be beneficial for the analysis of very large proteins, which is not much of an advantage for bacterial proteomes, which lack the large multidomain proteins seen in eukaryotic cells. A major downside of the bottom-up approach for PTM analysis is that, since the mixture of proteins undergoes digestion into peptides, the same detected peptide can belong to different proteins or isoforms. Furthermore, the connectivity between the peptides resulting from tryptic digestion is lost. In addition, the digestion step can lead to loss of sequence information, because they are too large or small to be detected by the instrument, or PTMs might not be identified due to stability issues on peptides.^18,19^ As lysine residues are commonly modified by PTMs, trypsin often cannot cleave after a modified lysine, which results in larger fragments and complicates the analysis.

The newer, emerging middle-down approach involves the use of either Glu-C or Asp-N proteases for limited digestions, which yield larger fragments commonly in the 3–9 kDa range, representing amino acid sequences of ∼20–100 residues (Figure 1).^20^ Glu-C is an enzyme that cleaves proteins C-terminally to aspartate or glutamate, while Asp-N cleaves proteins N-terminally to aspartate and to a lesser extent glutamate and cysteine. One advantage over the bottom-up method is reduced sample complexity due to larger peptide fragments, which increases the chance of identifying increased numbers of unique or lower abundance peptides. Another advantage is increased sequence coverage of proteins due to detection of larger fragments. Therefore, this technique can detect more PTMs and proteoforms, in part due to preservation of combinatorial PTMs that are contained on the same fragment.^21^ The advancement in fragmentation techniques, discussed below, have made the analysis of larger peptides more accurate and practical, exemplified by the study of the PTMs on eukaryotic histone tails.^22,23^ This technique may soon become more widespread and the preferred strategy for the characterization of PTMs.

In contrast to bottom-up or middle-down approaches, the top-down technique analyzes intact, not digested, proteins, thereby allowing for analysis of the entire PTM landscape of a protein (Figure 1). The idea behind the top-down method is that, from the mass spectrum of the entire protein, the intense peaks are fragmented, which indicate the sites of modifications and cleavages.^24,25^ Separation of proteoforms can be achieved by a variety of separation methods, like reverse-phase chromatography or 2D electrophoresis techniques.^26−28^ For PTM characterizations, this approach should identify the many different proteoforms that exist *in vivo*, which can provide information about the stoichiometry at each PTM site and the relative abundance of each proteoform. Sample preparation is simplified during this approach for most proteins, with no absolute requirement for chemical modifications, like reduction and alkylation, which can reduce the number of artifacts.^29^ While this would seem to be the preferred method of analysis of PTMs, there are challenges. Protein size is a limiting factor, where proteins <50 kDa will work better. There is also a need for more specialized MS equipment, which limits its widespread use. In addition, the entire protein is not always fragmented efficiently, which could lead to ambiguous PTM localizations or assignments.^30^ Finally, this approach is generally not considered high-throughput and may be better suited for the analysis and characterization of specific target proteins.^17^ Each technique has pros and cons for PTM analysis. Depending on the specific protein of interest and needs, any one of these three approaches could be effectively used to identify and characterize PTMs.

### MS-Based Proteomic Workflow

**Figure 2:**
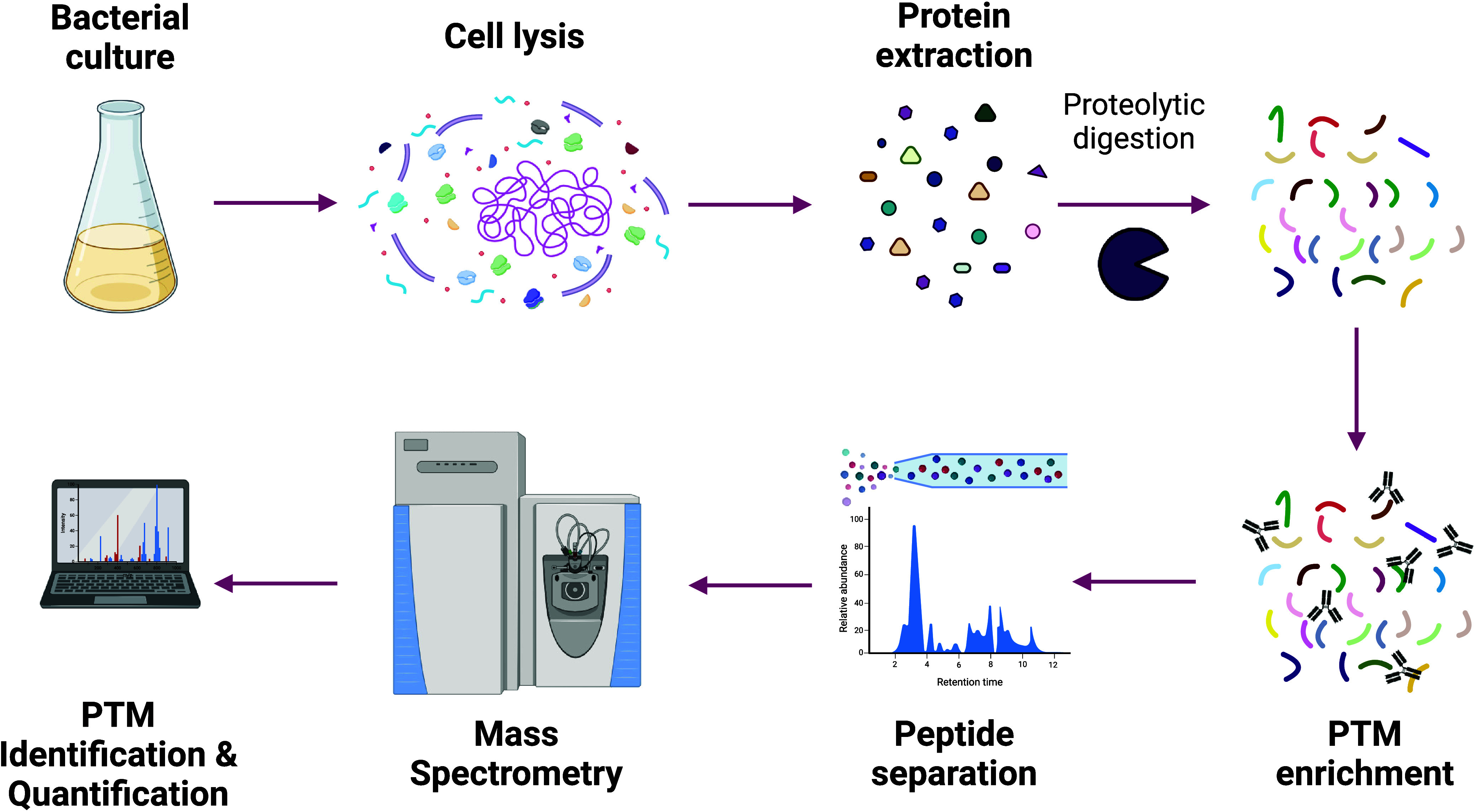
General MS workflow for PTM discovery. Bacterial cells are grown under desired conditions, and cells are lysed. Following extraction, proteins are digested into smaller fragments using an MS-compatible enzyme. PTMs are enriched using specific antiacyl antibodies, which are then further subjected to separation, often by liquid chromatography. Peptides are analyzed by MS techniques, and bioinformatic platforms are used for the identification and quantification of PTMs.

Global “acyl-omic” characterizations of bacteria generally consist of a similar basic workflow (Figure 2).^31^ First, the bacterial species of interest is grown under the desired growth conditions and cells are harvested. There are many different lysis techniques that can be used, but typically, protease and deacylase inhibitors, if available, are added to the lysis buffer to ensure identification of modifications. For a targeted analysis, the protein of interest is isolated by immunoaffinity purification.^12,14^ In either case, for bottom-up and middle-down approaches, following protein extraction, the next step is enzymatic digestion with trypsin, chymotrypsin, Glu-C, or other MS-compatible enzymes. Some workflows utilize an in-gel digestion, which allows for additional protein separation and a reduction in complexity of samples.^32^ As PTMs tend to have low abundance in bacteria, often in-solution digestion methods are better suited and preferred. For in-gel-based methodologies, the gel extraction step is often a place in the workflow where sample can be lost or contaminants or artifacts from the gel can complicate identification. For in-solution digestions, modified peptides are enriched using antiacyl antibodies, which are commercially available and are continually improving in quality and specificity, even for use in bacteria. To ensure a robust identification, mixtures of commercially available antibodies have been used successfully.^8,14^ The peptide approach has been useful for enrichment of modifications, so that the lysine residue is not buried in the protein and is more accessible to detection by the nonspecific antiacyl antibodies.^8^ Following isolation, the peptides are further fractionated, typically using liquid chromatography techniques.^33^

The samples are next prepared for MS analysis, often by desalting and removing detergents by use of filters or other column purifications.^34^ For PTM analysis, a high accuracy and sensitivity mass spectrometer, such as a Fourier-transform-based mass spectrometer, is required.^35^ Typically, a high-performance liquid chromatography module is configured with a nanoelectrospray ionization (nESI) source. Tandem MS analysis is carried out using several fragmentation strategies, including collision induced dissociation (CID), higher energy collisional dissociation (HCD), electron capture dissociation (ECD), and electron transfer dissociation (ETD).^36^ CID is a widely used technique where gaseous ions are collided with an inert gas to produce peptide fragments. HCD is similar to CID but uses a higher activation energy for better quality spectra. CID and HCD techniques may be useful for abundant and high stoichiometric PTMs, such as phosphorylation, but are not as useful for dynamic, low abundance modifications like acylations. ECD and ETD utilize electron-based fragmentation methods through neutralization of backbone protonation with thermal electrons or radical anions, respectively. ECD and ETD have large advantages over CID or HCD for detecting PTMs, because fragmentation is virtually independent of the amino acid sequence, which strongly influences fragmentation patterns in CID, and neutral losses are reduced, which simplify spectra and interpretation. Finally, numerous open source, freely available bioinformatic platforms exist, summarized in Table 1, that match identified peptides back to the protein, based upon a database search against the proteome of the species of interest and following removal of contaminants.

**Table 1:** Summary of Proteoform Identification Software Programs^a^

software program	key features
MASH Suite^37−39^	Interface to perform MS/MS search and manually validate MS/MS identifications
http://ge.crb.wisc.edu/software.html	
MetaMorpheus^40^	MS/MS search with PTM discovery and monoisotopic mass error notch search
http://github.com/smith-chem-wisc/MetaMorpheus	
MSPathFinder^41^	MS/MS search that identifies proteoforms with sequence graph and uses LC-data integration to improve monoisotopic mass determination
https://github.com/PNNL-Comp-Mass-Spec/Informed-Proteomics	
Proteoform Suite^42,43^	MS1-only to identify proteoforms by intact-mass observations and mass differences corresponding to modifications
http://github.com/smith-chem-wisc/ProteoformSuite	
TDPortal	MS/MS search against reference databases and biomarker search for truncated proteoforms
http://nrtdp.northwestern.edu/tdportal-request	
TopMG^44^	MS/MS tool for ultramodified proteoforms
https://toppic.sciencegateways.iu.edu/	
TopPIC^45^	MS/MS search against database with spectral alignment to determine unknown mass shifts
https://toppic.sciencegateways.iu.edu/	

a Modified with permission from ref (28). John Wiley & Sons, copyright 2019.

### Quantitative MS Approaches

The next questions that arise following the identification of site-specific PTMs are what is the abundance of this modification and how does it change in relationship to environmental conditions? There are three major quantification techniques for large-scale measurements of the abundance of modified proteins. Label-free quantification (LFQ) is routinely used to compare proteoforms among two conditions, which can be performed at the global level. LFQ measurements are based upon the measurements of ion intensity changes, like peak areas or peak heights from the chromatogram or spectral counting after MS/MS analysis.^46^ The advantage of this approach is that labeling agents are not required, which depending on the volume of the culture required could get costly. A disadvantage is that additional levels of fractionation are often required to reduce the sample complexity and identify lower abundance modifications.

Stable isotope labeling of amino acids in cell culture (SILAC) is based on labeling with isotopically labeled amino acids, both heavy and normal, that are provided individually to bacteria during growth.^47,48^ These isotopes are incorporated into proteins through translation and protein expression during growth. When two differentially grown cultures are mixed, the heavy and light labels are distinguished by mass differences caused by the different isotopes. The MS intensities of the two isotopic forms are then used to determine the differences in the abundance of the proteins or proteoforms between the examined conditions. Advantages of this technique are that it is easy to perform, minimizes quantitative error from handling a lot of samples in parallel, and does not require chemical reactions to modify proteins or peptides.^49^ A disadvantage is that the isotopically labeled amino acids can be expensive, and if working with large volumes of cells, this could become cost prohibitive.

Chemical labeling with small reporters enables multiplexing, with 4–8 samples possible with the use of isobaric tagging for relative and absolute quantification (iTRAQ)^50^ and up to 16 samples with tandem mass tag (TMT) labeling.^51^ These techniques can be used to identify and quantify proteins at the same time. For both techniques, a reagent is used to label primary amines in digested peptides from different samples. The samples are pooled and analyzed together for the remainder of the workflow. The isobaric nature of the tags leads to a combined signal of each peptide from all of the different labels at the MS1 level. Once subjected to fragmentation, the reporter ions, which are unique to each sample, are generated, with each one containing a different mass-to-charge ratio (*m*/*z*). From these ion intensities, relative quantification under the different conditions can be performed. Advantages of this approach are the time-saving multiplexing capabilities, the ability to achieve deep proteome coverage, and reproducibility, even between laboratories. The main disadvantage is that the labeling reagents are quite expensive, although some solutions to this barrier have been suggested.^52^ Quantitative proteomics is useful for the study of PTMs, because abundance changes under different conditions may hint toward functional significance. Therefore, these techniques can be used to prioritize acylated proteins for follow-up functional studies.^14^

## The Contribution of MS to Our Understanding of Lysine Acylations in Bacteria

As discussed above, MS-based proteomics has enabled the study of bacterial PTMs at the global level. Early proteomic analysis confirmed that PTMs are prevalent in bacteria and play important roles in central carbon metabolism, transcription, chemotaxis, and multicellular development, among others. Next, we discuss the advancements in our knowledge and understanding of bacterial physiology due to recent MS-based proteomic studies.

Over the past 5 years, there has been an increased investigation of the acetylomes of environmental bacteria, including those that live in extreme conditions. For example, *Shewanella baltica* is a Gram-negative bacterium that is capable of surviving low temperatures, including freezing, especially in aquatic environments. Their byproducts of metabolism cause the spoilage of aquatic products, including shrimp. As acetylated proteins are frequently found enriched in metabolic pathways, examination of the *S. baltica* acetylome provided a more complete understanding of the regulation of metabolic pathways, which could be useful for the design of new food preservatives. 2929 acetylation sites among 1103 acetylated proteins were identified, many of which were involved in metabolic processes.^53^ Importantly, enzymes involved in fatty acid metabolism, cold shock proteins, putrescine biosynthesis, and quorum sensing were acetylated. Further work is needed to fully understand the physiological significance of acetylation and to identify potential targets for food preservation. On the other extreme, the acetylome of the thermophilic *Thermus thermophilus* contains 335 sites on 208 proteins, which were mostly involved with metabolism or were ribosome associated.^54^ From this acetylome data, the role of acetylation on the enzyme 2-isopropylamine synthase, an enzyme involved in leucine biosynthesis, was examined. The four acetylation sites (K154, K332, K349, and K357) were mutated to the unacetylated (arginine, R) and acetylated (glutamine, Q) mimics. It was found that only the K332Q mutation inhibited activity, while the opposite mutation retained 70% of the activity. Thus, acetylation likely inhibits the activity of 2-isopropylamine synthase. The specific enzymes of acetylation are unknown, but a deacetylase was identified that removes the acetyl group, demonstrating that this modification is reversible and likely regulatory in nature.

*Nostoc flagelliforme*, a cyanobacteria that can survive extreme dehydration conditions, was analyzed with a focus on the role of lysine acetylation in response to dehydration stress. 2474 acetylation sites among 1060 proteins were identified, and LFQ analysis from different dehydration conditions indicated a global downregulation of acetylation in response to dehydration.^55^ Indeed, the differentially acetylated proteins were downregulated two to four times more across the different dehydration conditions compared to the control. Dehydration decreased lysine acetylation levels in certain metabolic pathways, such as the Calvin cycle and the ROS scavenging system, indicating that acetylation may regulate photosynthesis and abiotic stress tolerance.

*Dehalococcoides mccartyi*, a bacterium that breaks down organohalides for growth and energy, has been used to break down organohalide contaminants in polluted groundwater and soil. To gain insights into the survival mechanisms at limited concentrations of organohalides, an acetylome analysis of *D. mccartyi* during both exponential and stationary growth was performed. There were 192 acetylated peptides detected in both growth phases.^56^ During the stationary phase, there was an increased number of acetylated proteins involved in xenobiotic biodegradation and metabolism. An interesting finding is that the twin-arginine translocation protein TatA, which translocates folded proteins across the membrane,^57,58^ was acetylated exclusively in stationary phase. This acetylation pattern is consistent with this system’s role in energy metabolism and the requirement of a functional organohalide respiration complex. It was proposed that TatA acetylation may reduce Tat-dependent transport, which, as this process is energetically demanding, may be important in suboptimal growth conditions. This was the first report of TatA acetylation in any bacterium, which adds a new regulatory layer to this broadly conserved system.^59^

*Azorhizobium caulinodans* contains 2302 acetylation sites on 982 proteins, and as with many bacterial species, acetylated proteins were involved in energy metabolism.^60^ In particular, acetylated proteins were enriched in the TCA cycle, ribosomes, metabolism of glycerol and lipids, glucose, pyruvate metabolism, and bacterial chemotaxis. One acetylated protein, the chemotaxis master regulator CheY, was further examined. CheY is acetylated at five sites (K16, K27, K100, K110, and K118), and sequence alignment with orthologs from *E. coli* and *Salmonella* Typhimurium revealed that acetylation at site 110 is conserved. A Δ*cheY1* deletion and *cheYK110Q* mutant resulted in significantly impaired swimming motility; however, the *cheYK110R* mutation had an intermediate phenotype, which indicates that acetylation of K110 may disrupt chemotactic motility. It was proposed that acetylation may regulate the binding of CheY to flagella-associated proteins. Furthermore, deletion of deacetylase Δ*AZC_0414* displayed significant increases in chemotactic motility, which likely indicates that AZC_0414 regulates chemotaxis by deacetylating CheY or other important motility proteins.

*Deinococcus radiodurans* is one of the most resistant organisms to ionizing radiation and oxidative stress.^61^ Characterization of the exponential phase acetylome revealed 4364 acetylation sites on 1410 proteins, accounting for 45.7% of the theoretical proteome.^62^ Many of the proteins of this bacterium are undefined and uncharacterized, but acetylated proteins were enriched in amino acid transport and metabolism, translation, and energy production and conversion. Especially interesting for *D. radiodurans* was the observation that many DNA damage repair pathways contained acetylated proteins, suggesting the possibility that acetylation is an important regulatory mechanism of DNA repair systems. It would be interesting to further analyze these acetylated proteins under ionizing radiation exposure or oxidative stress to explore their exact role in these important survival pathways.

There are now commercially available, high-quality antibodies directed against many different lysine acylation modifications, including succinylation, propionylation, and malonylation, which have enabled their study. The next most studied lysine modification is succinylation, a PTM that results in a charge reversal and adds a negatively charged moiety. Succinylome analysis of the periodontal pathogen *Porphyromonas gingivalis* led to the identification of 345 sites on 233 proteins, accounting for 11.1% of the proteome.^63^ Many important virulence factors, including gingipains, fimbriae, and lipopolysaccharide biosynthesis proteins, were modified, suggesting that succinylation may also regulate virulence or other essential processes. The succinylome of *D. radiodurans* was also characterized.^64^ There were 492 sites present on 270 proteins. The most significantly enriched category was related to nucleic acid metabolism, which is different from most reports that list carbon metabolism and ribosomes associated as the top categories. As seen with acetylation, 28 of these proteins were involved in DNA repair or stress responses. To further explore the role of succinylation in double strand break repair, two succinylated proteins, the protease PprL and the strand annealing protein DbrB, were analyzed. Mutants mimicking the negatively charged succinylated form (glutamate substitution, E), unmodified, positively charged form (R), and neutral charge (alanine, A) were created. PprLK185E led to a decrease in enzymatic activity, while the R mutant had no effect, suggesting that succinylation is a negative regulator of PprL activity. Similarly, mutation of K103 or K108 of DbrB to glutamate or alanine resulted in an inhibition of DNA binding activity, again suggesting that succinylation negatively affects the enzymatic activity. Succinylome analysis of the commensal bacterium and opportunistic pathogen *Staphylococcus epidermidis* led to the identification of 1557 sites on 649 proteins.^65^ It was observed that, among species where the succinylomes were reported, there was a 31.4% overlap with *E. coli*(66) and *Vibrio parahemolyticus*,^67^ suggesting that succinylation represents a conserved regulatory mechanism for some proteins. The succinylated proteins were enriched in translation and central carbon metabolism, as was often observed. With the catalogs of succinylated proteins being continually published, the next step will be to perform mutational analyses with the mimic mutations, both *in vivo* and *in vitro* to fully understand the significance of succinylation. Also, identifying enzymes with succinylase and desuccinylase activities is crucial. It has been suggested that, in *E. coli*, the deacetylase CobB also has desuccinylase activity,^68^ but it is not known if this is a conserved property among all orthologs.

Large-scale analyses of less common acyl modifications are starting to be performed. The investigation of acyl modifications in *B. subtilis* demonstrated that 35.8% of all proteins are modified, with 2536 acetylated, 2150 succinylated, 4723 propionylated, and 3001 malonylated sites. Furthermore, 420 acylated sites were identified in common between all four modifications, and these proteins were involved in important pathways, such as amino acid metabolism, translation, nucleic acid metabolism, and glycolysis.^69^ These findings raise further questions and possible avenues of study to explore the potential relationship between the four PTMs and the possibility of crosstalk among them, for which MS-based proteomics will be essential.^70^ Another study explored the *B. subtilis* phosphoproteome and acetylome in different growth media including nutrient-limited and rich conditions. Over 1600 MS runs were analyzed, and 3159 proteins, with 1085 phosphorylation and 4893 lysine acetylation sites, were characterized, which covered 75% of the theoretical proteome.^71^ These large, comprehensive data sets are a valuable resource to the field and can be used to identify new acylated proteins to examine the physiological significance of lysine acylation.

The acetylome and butyrylome of the spore-forming, Gram-positive bacteria *Clostridioides acetobutylicum* was characterized using a quantitative proteomic method involving stable isotope dimethyl labeling,^72,73^ during exponential, transitional, and stationary phases. 254 proteins with 458 lysine acetylation sites and 373 proteins with 1078 lysine butyrylation sites were identified. Lysine butyrylation was increased in the transitional phase, while global acetylation remained constant. As one of the species beneficial to humans due to the production of the biofuel butanol, this species has additional interest in terms of the possibility of engineering a superior butanol-producing strain. The master regulator of sporulation Spo0A was butyrylated at K45 and K217. It was demonstrated that butyrylation at K217, which occurs in the helix-turn-helix DNA binding motif, inhibits binding of Spo0A to its own promoter but not to that of the *sol* operon, which encodes butanol formation-related proteins.^73^ As these studies were performed *in vitro* by electrophoretic mobility shift assays (EMSAs), it would be interesting to examine the effects of *spo0AK45Q* and *spo0AK217Q* mutants on transcription using *in vivo* techniques such as quantitative real-time PCR or RNA-seq to examine the biological significance of these modifications.

In the intracellular parasite *Brucella abortus*, five different acyl modifications were studied: 2-hydroxyisobutyrylation, succinylation, crotonylation, acetylation, and malonylation.^74^ The most common modification was 2-hydroxyisobutyrylation, and the least common was malonylation. Common among the five PTMs were 548 proteins, and most proteins (≥96%) contained at least one modification. Many virulence proteins were modified by one or several PTMs, which included proteins that are important for cell division, invasion, stress responses, immune evasion, and intracellular survival. Due to the high number of virulence factors identified as modified in this study, it was proposed that these acylations would improve the ability of *B. abortus* to survive intracellularly. These ideas have not been experimentally validated. The malonylome of *Staphylococcus aureus* has also been explored, revealing 440 sites on 281 proteins during stationary phase.^75^ Malonylated proteins are largely enriched in glycolysis, central carbon metabolism, and translation. Malonylation is poorly understood, but emerging evidence suggests that this modification may also be regulatory in nature. Tools to study this PTM are lacking, and future studies should aim to perform *in vitro* analysis to probe the physiological significance of this acyl modification. The findings from studies discussed in this section and the remaining sections are summarized in Table 2.

**Table 2:** Summary of 2018–2023 Acylome Studies^a^

species	modification	sites modified	proteins modified	growth conditions	ref
*A. caulinodans*	Acetyl	2302	982	Stationary phase, minimal media	(51)
*A. hydrophila*	Acetyl	3189	1013	OD of 1.0, rich media	(70)
	Succinyl	2174	666	OD of 1.0, rich media	
*B. abortus*	Acetyl	4317	1191	Exponential phase	(65)
	Succinyl	5825	1293	Exponential phase	
	Hydroxyisobutyryl	6953	1336	Exponential phase	
	Crotonyl	5709	1290	Exponential phase	
	Malonyl	1693	612	Exponential phase	
*B. burgdorferi*	Acetyl	61	52	Exponential phase	(81)
	Acetyl	104	64	Stationary phase	
*B. pertussis*	Acetyl	2061	761	Stationary phase	(83)
*B. subtilis*	Acetyl	2536	866	Stationary phase	(60)
	Succinyl	2150	634	Stationary phase	
	Propionylation	4723	1214	Stationary phase	
	Malonyation	3001	973	Stationary phase	
	Acetyl	4893	1277	Minimal and rich media	(44)
	Acetyl	1772	826	Biofilm	(79)
*C. acetobutylicum*	Acetyl	458	254	Exponential, transitional, stationary phases	(64)
	Butyryl	1078	373	Exponential, transitional, stationary phases	
*D. mccartyi*	Acetyl	192	N/A	Exponential/stationary phase	(47)
*D. radiodurans*	Acetyl	4364	1410	Stationary phase	(52)
*D. radiodurans*	Succinyl	492	270	OD of 1.0	(55)
*E. tarda*	Acetyl	1511	589	OD of 1.0, rich media	(69)
	Succinyl	2353	692	OD of 1.0, rich media	
*F. tularensis*	Acetyl	1178	280	Chemically induced acetylation	(99)
*N. flagelliforme*	Acetyl	2474	1060	Dehydration stress	(46)
*P. aeruginosa*	Acetyl	1102	522	Citrate, glucose, glutamate, succinate	(84)
	Succinyl	1522	612	Citrate, glucose, glutamate, succinate	
*P. gingivalis*	Acetyl	1112	438	OD of 1.1	(72)
	Succinyl	345	233	OD of 1.0	(54)
*S. aureus*	Succinyl	3260	799	Stationary phase	(67)
	Acetyl	7935	1710	Stationary phase	(68)
	Malonyl	440	281	Stationary phase	(66)
*S. baltica*	Acetyl	2929	1103	OD of 0.7, rich media	(44)
*S. coelicolor*	Acetyl	3981	1522	ScCobB1 mutant	(67)
	Succinyl	5982	1510	ScCobB2 mutant	
*S. epidermidis*	Succinyl	1557	649	Stationary phase	(56)
*S. mutans*	Acetyl	617	445	Biofilm	(80)
*S. pneumoniae*	Acetyl	653	392	OD of 0.6	(96)
*S.* Typhimurium	Acetyl	1259	631	Ciprofloxacin susceptible and resistant strain	(98)
*T. thermophilus*	Acetyl	335	208	Stationary phase	(45)
*V. alginolyticus*	Acetyl	2883	1178	Exponential phase	(90)
	Acetyl	1315	689	Bile salt stress	(91)
	Succinyl	2082	671	OD of 1.0	(92)
*V. cholerae*	Acetyl	3402	1240	Midexponential and stationary phase	(88)
*V. mimicus*	Acetyl	1097	582	OD of 1.0	(93)
*V. vulnificus*	Acetyl	6626	1924	LB, alkaline peptone, artificial seawater	(89)
*Y. pestis*	Acetyl	1491	288	Mammalian host conditions	(87)
	Acetyl	414	86	Flea vector conditions	

a OD, optical density; LB, lysogeny broth.

## Proteomic Contributions to Our Understanding of PTM Crosstalk

**Figure 3:**
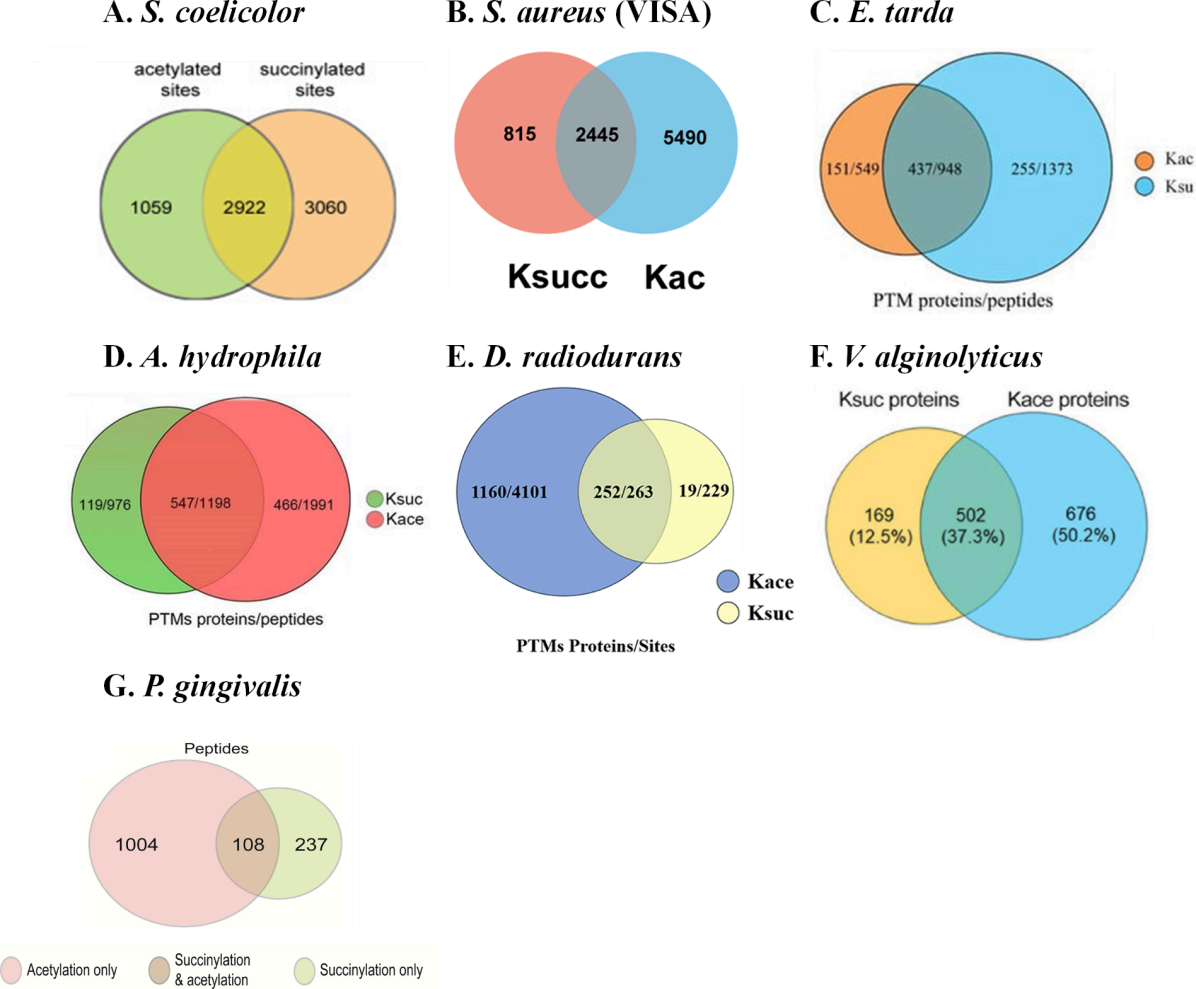
Summary of unique and common acetylated and succinylated sites and proteins in various bacteria. Venn diagrams illustrating the overlapping acetyl and succinyl sites in (A) *S. coelicolor*, adapted from ref (76) under the Creative Commons Attribution (CC BY 4.0) license. (B) VISA, adapted from ref (77) under the CC BY 4.0 license. (C) *E. tarda*, adapted from ref (78) under the CC BY 4.0 license. (D) *A. hydrophila*, adapted from ref (79) under the CC BY 4.0 license. (E) *D. radiodurans*, adapted from ref (62) under the CC BY 4.0 license. (F) *V. alginolyticus*, adapted from ref (80) under the CC BY 4.0 license. (G) *P. gingivalis*, modified with permission from ref (81). John Wiley & Sons, copyright 2020.

There is a definite relationship between lysine modifications, especially those noted for acetylation and succinylation. In *Streptomyces coelicolor*, most acetylated proteins were also succinylated; specifically, 2511 sites which accounted for 63.1% and 50.0% of the identified sites in the acetylome and succinylome, respectively. These dually regulated proteins were enriched in the TCA cycle and protein translation and sometimes occurred at the same site. Furthermore, analysis of mutant strains lacking the deacetylase *cobB1* or desuccinylase *cobB2* suggested that both enzymes were bifunctional, working as both a deacetylase and desuccinylase, suggesting a possible competitive relationship. The factors that influence this relationship and determine which activity predominates for these enzymes are currently unknown and could reveal a complex regulatory relationship between them.^76^ Another example of a bifunctional enzyme was reported in vancomycin-intermediate *Staphylococcus aureus* (VISA), in which the sirtuin SaCobB was reported to have both deacetylase and desuccinylase activities. In VISA, there were 3260 lysine succinylation sites among 799 proteins and 7935 lysine acetylation sites in 1710 proteins identified. Interestingly, 75% of succinylated sites were found to be acetylated, which agrees with the study on *S. coelicolor* (Figure 3).^76^ The dually modified proteins were mainly involved in translation, aminoacyl-tRNA synthesis, and the glycolysis/gluconeogenesis pathways.^77^ Understanding the physiological significance of both modifications, identifying the mechanisms of acylation and deacylation, and deciphering the timing and chemical/biological cues regulating these modifications are of great interest.

Understanding the relationship between acetylation and succinylation is important for the field going forward, especially in the context of pathogenic bacteria. *Edwardsiella tarda* is a commonly found aquatic pathogen that rarely infects humans. The overuse of antibiotics has resulted in high rates of antibiotic resistance, especially to ampicillin and oxytetracycline. In *E. tarda*, 589 proteins were acetylated and 692 proteins succinylated.^78^ Interestingly, antibiotic resistance genes, such as efflux pumps, porins, and drug targets, were frequently acylated, suggesting a role of these modifications in the regulation of resistance traits. Regarding general metabolism, there was a significant overlap between proteins modified by acetylation and succinylation, with 437 proteins containing 948 sites, often modifying the same residue (Figure 3). Enrichment analysis of this set of proteins indicated that many are involved in carbon metabolism, translation, RNA degradation, glycolysis, and the TCA cycle. Further study is required to fully evaluate the role that these acylations play in virulence and drug resistance in *E. tarda*.

Acetylation analysis of another fish pathogen, *Aeromonas hydrophila*, revealed 3189 sites on 1013 proteins, which mostly functioned in essential metabolic pathways.^82^ Succinylome analysis led to the identification of 2174 sites on 666 proteins.^83^ 1198 sites on 547 proteins were common for both acetylation and succinylation (Figure 3). The most common functions of these proteins were those involved in the TCA cycle, pyruvate metabolism, and glucose breakdown or synthesis. Further examination of these dually modified proteins led to the study of the acylation of S-ribosyl homocysteine lyase (LuxS) at K165. Substitution mutations of lysine were constructed, which changed this residue to glutamate, arginine, or glutamine, mimicking succinylation, deacetylation, and acetylation, respectively. The analysis of enzymatic activity showed a reduction of LuxS activity when acetylated and increased activity when succinylated, suggesting that these two modifications can oppose each other’s activity.^83^ This is one of the few explorations into the biological significance of these dual modifications on a specific protein, and further research in this capacity is required. This data suggest a competitive nature between these two PTMs when they share a common site, and the next step is to uncover conditions and mechanisms that influence this balance.

MS-based acetylome and succinylome analysis of *P. gingivalis* showed that >60% of succinylated proteins were also acetylated, and 108 of the lysine acetylation sites in 83 proteins were identical for succinylation (Figure 3). The dually modified proteins were enriched in aspartate and glutamate catabolism and associated with the ribosome.^81^ Aspartate and glutamate catabolism is essential for *P. gingivalis* survival because they produce energy and toxic metabolic end products.^84,85^ Lysine modified proteins may play important roles in virulence, as important virulence factors are acetylated or succinylated, including adhesins, gingipains, antioxidants, and proteins involved in amino acid catabolism.^81,86^ The observation of crosstalk is becoming more widespread, and understanding the intricacies of the relationships among modifications will be the next horizon in the study of bacterial PTMs.

## MS-Based Proteomics Adds a New Layer to Biofilm Regulation

**Figure 4:**
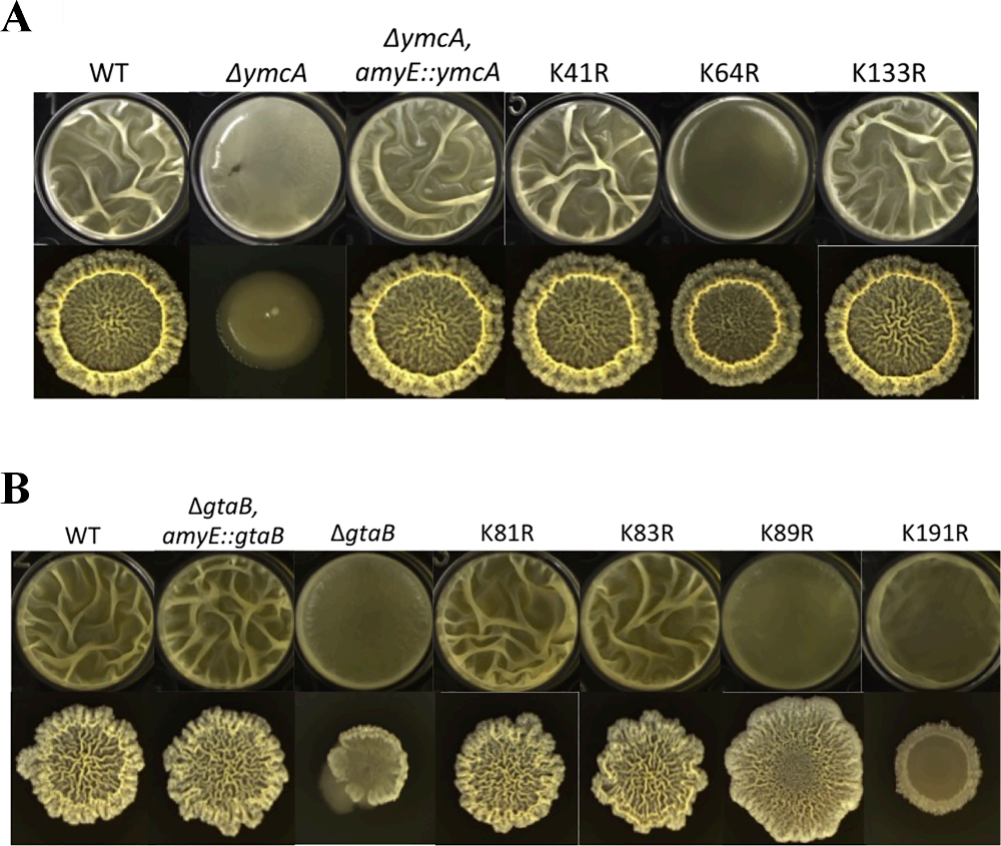
Biofilm phenotype of deacetylation mimic mutants of YmcA (RicA) and GtaB. *B. subtilis* variants with the noted acetylation sites mutated to the deacetylation mimic arginine. The top row shows pellicle formation at the air–liquid interface, and the bottom row shows the biofilm colony morphology on solid media. (A) The *ymcAK64R* mutant had a severe defect in biofilm formation, as evidenced by less wrinkling on the pellicle and a smaller sized colony, which suggests that acetylation of YmcA at K64 is regulatory and required for this process. (B) The *gtaBK89R* mutant is also defective in biofilm formation. These mutants had featureless pellicles and a larger colony size with less pronounced wrinkles. This suggests that the acetylation of K89 is required for proper GtaB function. Modified from ref (90) under the Creative Commons Attribution (CC BY 4.0) license.

In nature, nearly every bacterial species can assemble into highly structured, multicellular communities termed biofilms. Bacteria in a biofilm secrete an extracellular polymeric substance (EPS), which is made of polysaccharides, proteins, and extracellular DNA.^87^ Clinically, biofilms are extremely difficult to treat due to their increased resistance to both innate and adaptive immune responses^88^ and tolerance to antibiotics.^89^ This prompted various researchers to examine the role of protein acetylation in biofilm formation. In one study of the *B. subtilis* acetylome during biofilm formation, two key regulatory proteins were identified as acetylated, a biofilm regulatory protein RicA (formerly known as YmcA) and GtaB (UTP-glucose-1-phosphate uridylyltransferase), which is involved in the formation of EPS. When the lysine acetylation sites were mutated to mimic the unacetylated form, *ymcAK46R* and *gtaBK89R* strains exhibited severe biofilm defects (Figure 4).^90^ From the examination of the acetylome data, important regulatory proteins were identified for further study, which resulted in a new dimension added to the regulation of biofilms. It is possible that a specific acetyltransferase is induced under biofilm conditions that regulate specific proteins needed for biofilm formation. As *B. subtilis* contains 50 annotated acetyltransferases, it is conceivable that one of these represents a biofilm specific enzyme.

Characterization of the acetylome of *Streptococcus mutans* biofilms in comparison to planktonic growth demonstrated that there were 445 proteins with 617 acetylation sites, of which many where quantified using TMT labeling and high-resolution MS. There were ∼100 differentially acetylated proteins, and of interest were the glycosyltransferases. These enzymes are involved in the production of EPS from dietary sucrose and were less acetylated during biofilm growth, suggesting an off-switch mechanism to regulate their activity.^91^ This hypothesis has not been experimentally confirmed; therefore, further studies are required. MS-based proteomics can supplement and expand our understanding of bacterial developmental fates, and these experiments are worth expanding to other species, especially human pathogens.

## MS-Based Proteomics Contributions toward Bacterial Pathogenesis

As large data sets from MS-based studies became more available, the potential role in growth rate determination and pathogenesis led to increased exploration and characterization of lysine acetylation in pathogenic species.^9,13^ The lysine acetylome was characterized during midexponential and stationary phase of the causative agent of Lyme’s disease *Borrelia burgdorferi*. In this species, only 5% of proteins were acetylated, and the majority were found in central metabolism, which has been observed for many other bacteria.^92^ 61 acetylated peptides from 52 unique proteins were identified during exponential phase, and 104 acetylated peptides from 64 proteins were identified during stationary phase. Most of these proteins overlapped between the two growth phases, with only 4 unique to midexponential phase and 16 unique in stationary phase. The acetylated proteins were largely involved in carbon metabolism, motility and chemotaxis, transport, and DNA processes. By characterizing the acetylome of Δ*ackA* strains, which lack donors, acetyl phosphate (Ac-P) and acetyl-CoA (Ac-CoA), and Δ*pta*, which lacks only Ac-CoA, it was determined that Ac-P was the primary acetyl donor in this species, in agreement with other bacterial species.^11,93^

The increasing problem of multidrug-resistant (MDR) pathogens and lack of effective antibiotic treatment pushed research to identify new potential targets within the bacterial cell. One such target could be modified proteins: key virulence factors, essential proteins, or enzymes of acetylation. In *Bordetella pertussis*, the causative agent of whooping cough, an LFQ acetylome analysis comparing a wild-type and mutant strain lacking the lysine deacetylase *bkd1* was performed. In *B. pertussis*, there were 761 unique acetylated proteins, and 198 were deacetylated by Bkd1. Some important virulence factors, such as the master regulator BvgA of the BvgAS two-component system, had increased acetylation in the *bkd1* mutant strain, but most other known important factors did not. This suggests that Bkd1 is not important for the regulation of virulence, and in agreement, it did not have any influence on survival in human macrophages *in vitro*. There was substantial evidence that Bkd1 regulates housekeeping functions like metabolism and homeostasis.^94^ From this quantitative mass spectrometry analysis, Bkd1 was identified as a bona fide deacetylase, substrates were identified, and its role in housekeeping functions but not bacterial virulence was established. This work suggests that Bkd1 would not be an effective target for novel drug design.

**Figure 5:**
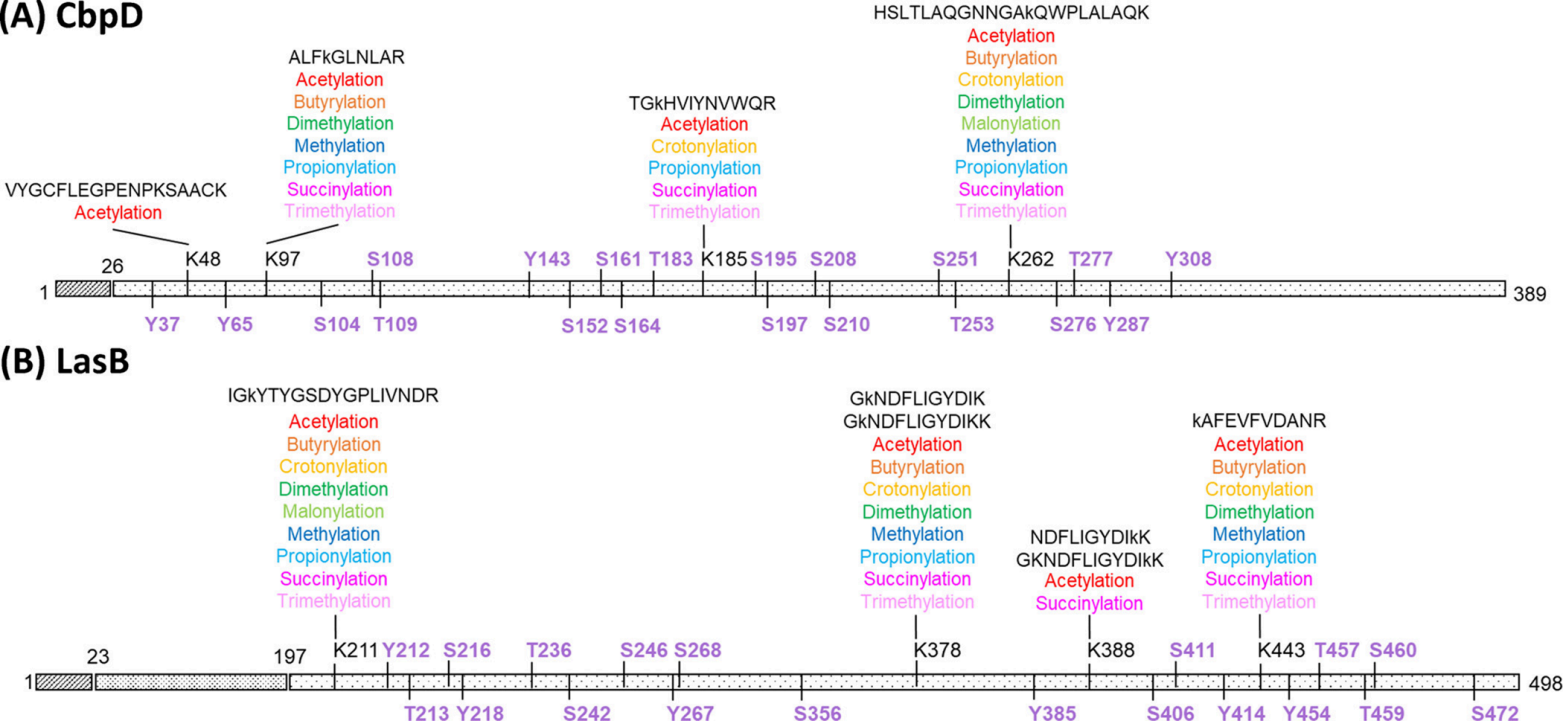
Acyl analysis of two virulence factors of *P. aeruginosa*. The virulence factors CbpD (A) and LasB (B) with their identified PTMs are noted. The possible acyl modifications of lysine residues include acetylation, butyrylation, crotonylation, malonylation, propionylation, and succinylation. Also noted are the lysine modifications of mono-, di-, and trimethylation and serine, threonine, and tyrosine phosphorylation sites. Reprinted from ref (96). Copyright 2019, American Chemical Society.

In *Pseudomonas aeruginosa*, the acetylome and succinylome were characterized following growth on four different carbon sources: citrate, glucose, glutamate, and succinate.^95^ Overall, 612 succinylated and 522 acetylated proteins were identified, with citrate containing the most succinylated proteins and acetylated proteins being equivalent among the conditions. There were 622 lysine sites among 321 proteins that could be modified by either acetylation or succinylation at the same site, suggesting potential crosstalk between these modifications. Virulence proteins were identified that contained both modifications. For example, the multidrug efflux pump protein MexA, which is part of the MexAB-OprM system, was succinylated and acetylated at multiple sites. It was proposed that succinylation of K136 may modify the structure and functionality of the pump. Other virulence factors, such as proteins involved in chemotaxis, detoxification, and persistence, were shown to be modified by one or more PTMs. Follow-up studies on the virulence factors CbpD, a chitin-binding protein, and the elastase LasB revealed that they were modified by acetylation and succinylation.^96^ The analysis was expanded to examine nine total modifications, including acetylation, butyrylation, crotonylation, malonylation, propionylation, succinylation, methylation, dimethylation, and trimethylation. It was found that individual sites were modified by one to all nine modifications (Figure 5). 2D gel electrophoresis, followed by MS analysis, revealed that there were at least 32 proteoforms of CbpD and three for LasB in the cell. CpbD and LasB can be found intracellularly and excreted into the extracellular environment. Interestingly, some modifications were detected only either intracellularly or extracellularly, suggesting that different proteoforms may be compartment specific. These findings suggest that acetylation and other acyl modifications play a key role in virulence, and it would be valuable to further explore these modified proteins or the enzymes involved to possibly identify new therapeutic targets. As MDR *P. aeruginosa* strains continue to emerge and treatment options become more limited, the exploration of alternative drug targets becomes even more important.^97^

The acetylome of *Yersinia pestis*, a flea-borne pathogen and the causative agent of the three bubonic plague pandemics in history, was analyzed under conditions to mimic its two natural niches, mammalian hosts and the flea vector, in order to understand the role of protein acetylation in adaptation to different environments. Acetylation frequently occurs in *Y. pestis*, with 32.6% of the proteome containing this PTM. Overall, more acetylated proteins were identified in mammalian conditions, corresponding to 288 (20.6%) acetylated proteins compared to 86 (6.2%) in the flea environment.^98^ Most of the acetylated proteins found in these mammalian host conditions were involved in stress and damage responses. From these acetylome analyses, it was discovered that SlyA was acetylated at K73, which was deacetylated by the sirtuin CobB. SlyA is an important transcriptional regulator of virulence and biofilm formation. It was found that SlyA acetylation inhibited DNA binding and significantly increased biofilm formation, suggesting that acetylation acts to relieve the repression of specific genes, such as *hmsT* and *hmsD*, which are involved in biofilm formation. Thus, acetylome analysis uncovered another layer of regulation of an important virulence factor for *Y. pestis*.^98^

The *Vibrionaceae* family of bacteria is a common cause of gastroenteritis in humans and marine diseases in fish. Recently, the acetylomes of these pathogens have been extensively explored. In *Vibrio cholerae*, the causative agent of cholera, 3402 acetylation sites on 1240 proteins were identified, including 189 acetylation sites on 68 proteins corresponding to 33% of known virulence factors.^99^ Acetylated proteins were found to be involved in several additional important processes, such as motility, chemotaxis, and biofilm formation. This suggests that acetylation may be important for the regulation of virulence in *V. cholerae*,^99^ and further exploration of the physiological significance of these modifications is warranted. *Vibrio vulnificus* causes gastroenteritis but can also cause wound infections, which could be fatal in immunocompromised patients. The acetylome of a *V. vulnificus* strain that was isolated from a patient with necrotizing fasciitis was examined.^100^ Global acetylome analysis revealed 6626 acetylation sites on 1924 proteins, with many acetylated proteins involved in central carbon metabolism and virulence factors, including those which promote adherence, host cell cytotoxicity, and possibly tetracycline resistance.

The economic losses due to fish disease account for approximately 150 million USD annually, and one of the responsible pathogens is *Vibrio alginolyticus*, which not only infects fish but also causes ear and wound infections in humans. Acetylome analysis identified 2883 acetylated sites on 1178 proteins, which were involved in various metabolic processes, and 63 of the acetylated proteins were involved in virulence and host–pathogen interactions.^101^ To further study *V. alginolyticus* pathogenesis, an acetylome analysis was performed during bile salt stress, a condition that would be encountered in the gut environment. Specifically, 22 virulence factors were found to be acetylated, including those involved in chemotaxis and motility, adherence, toxin production, and secretion systems. Similar to the previous untreated *V. alginolyticus* analysis,^101^ acetylation under bile salt stress revealed that acetylated proteins were involved in different metabolic pathways with 240 proteins overlapping.^102^ Furthermore, succinylome analysis of *V. alginolyticus* showed a significant overlap of acetylation and succinylation sites in 503 (37.3%) proteins, suggesting widespread crosstalk between both acylations. Succinylation also plays a significant regulatory role in central metabolic pathways and bacterial virulence. Overall, there were 2082 succinylation sites on 671 proteins and 50 succinylated virulence factors, 40 of which were predicted to interact.^80^ The specific roles that both succinylation and acetylation play in the regulation of bacterial virulence in *V. alginolyticus* will be interesting for future research. The aquatic animal pathogen *Vibrio mimicus* is another reason for economic losses in the food industry and causes a threat to food safety, leading to symptomatic gastroenteritis in humans who consume contaminated food. An acetylome analysis of *V. mimicus* identified 1097 acetylation sites in 582 acetylated proteins, and further bioinformatics analyses indicated that acetylated proteins are mainly found in energy metabolism, secondary metabolite biosynthesis, and various pathogenic processes.^103^ Interestingly, the pathways enriched for acetylated proteins are conserved among Vibrio species, including *Vibrio cholerae*, *V. alginolyticus*, *V. parahemolyticus*, and *V. vulnificus*.^100,101,104,105^ This suggests that acetylation may be an important conserved regulatory process in the basic physiology of bacterial pathogenesis in the entire genus.

The high diversity of *Streptococcus pneumoniae* serotypes makes the present pneumococcal vaccines less efficient, which requires additional research on the regulation of bacterial metabolism and virulence. Analysis of the acetylome led to the identification of 653 lysine acetylation sites on 392 proteins, which may regulate diverse metabolic pathways, such as energy metabolism, translation, central metabolism.^106^ Acetylated virulence factors were also identified, including those associated with capsule polysaccharide synthesis and assembly, adherence, invasion, and evasion of host immunity. One interesting finding was that nine proteins involved in capsule polysaccharide (CPS) biosynthesis were acetylated. CPS is essential for virulence and survival and is precisely regulated depending on the host environment during infection.^107^ These findings suggest that lysine acetylation may be a critical regulatory factor for capsule production, which is the target of our current vaccines. A full understanding of this process may aid in future vaccine development.

As with many bacterial infections, drug resistance among *Salmonella* infections is an increasing problem, especially resistance to first-line fluoroquinolones. A comparative mass spectrometry acetylome analysis was performed using ciprofloxacin susceptible and resistant *Salmonella* Typhimurium strains. In total, 1259 lysine acetylation sites were identified in 631 proteins, with the majority of differentially acetylated proteins in basic metabolic processes.^108^ Additionally, 14 acetylated proteins were related to antibiotic resistance, suggesting that acetylation may be important not only for establishment of infection in the host environment but also for survival of therapeutic interventions.

Acetylome analysis of *Francisella tularensis* ssp. *novicida*, the causative agent of tularemia, revealed 1178 acetylation sites on 280 proteins.^109^ This analysis was performed using chemical acetylation by AcP incubation to increase the number of identifications. It is unclear whether these chemically induced acetylations occur under biological conditions and are meaningful. The acetylated proteins were mainly involved in metabolism, transcription, and translation, as has been observed for most bacterial species. The chitinases were acetylated at multiple sites and were selected for additional characterization. Chemical acetylation with Ac-P led to a decrease in endochitinase activity, suggesting that acetylation interferes with enzyme–substrate dynamics. Chitinases A and B are negative regulators of biofilm formation.^110^ This is of interest in view of the exploration of new treatments for bacterial biofilm-based infections. For example, the use of chitinases with acetylation inhibitors may stimulate biofilm degradation and assist conventional antibiotics in infiltrating the infection site. This is another example of how the understanding of the biological significance of PTMs could lead to novel drugs with new mechanisms of action.

## MS Contributions to Our Understanding of the Mechanisms of Bacterial Acetylation

**Figure 6:**
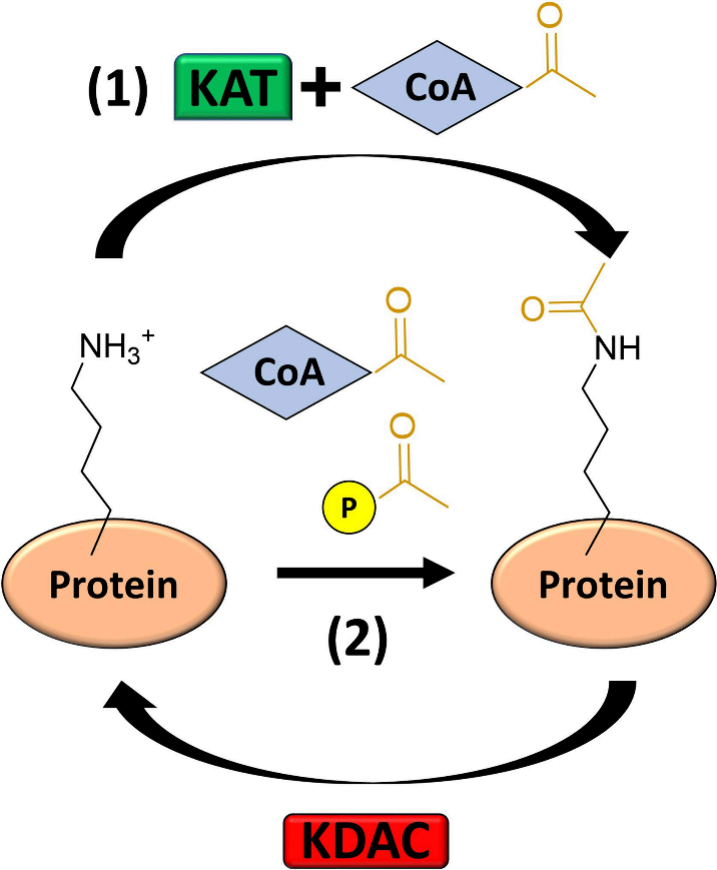
Bacterial mechanisms of acetylation and deacetylation. (1) Acetylation is carried out by lysine acetyltransferases (KATs), which catalyze the transfer of the acetyl group from Ac-CoA to the target lysine residue. Alternatively, lysines can be nonenzymatically acetylated, predominantly by using Ac-P as the donor. (2) These reactions are reversible by the action of the lysine deacetylases (KDACs), which in bacteria are mostly NAD^+^-dependent sirtuins. Reproduced from ref (11) under the Creative Commons Attribution (CC BY 4.0) license.

Proteomics has largely contributed to our understanding of the mechanism of acetylation in bacteria.^11,12,14,111,112^ In bacteria, there are enzymatic and nonenzymatic mechanisms of acetylation, which were eloquently established over the last 20 years. MS-based proteomics has contributed to our fundamental understanding of both, especially for the nonenzymatic mechanism.^12,14,112^ The enzymatic mechanism involves a lysine acetyltransferase (KAT), typically a Gcn5-N-acetyltransferase (GNAT) family member, that removes the acetyl group from the donor molecule acetyl-CoA and transfers it to the amino group of the target lysine side chain (Figure 6).^113,114^ The first example of enzymatic acetylation discovered in bacteria was the identification of the enzymes involved in the regulation of acetyl-CoA synthetase in *Salmonella enterica*,^115,116^ which was later shown to be true of many of the AMP-intermediate forming enzymes.^10,117^ The second mechanism of acetylation is nonenzymatic, whereby the local environment surrounding the target lysine residue influences the protonation status of the amino group, making it a better nucleophile to attack the carbonyl carbon of Ac-P.^118,119^ Acetylation by either mechanism can be reversed by the action of the deacetylases, which in bacteria, the most widespread are the NAD^+^-dependent sirtuins.^120^ Global acetylation analysis in *E. coli* revealed that the predominant mechanism of acetylation is nonenzymatic, whereas the known KAT YfiQ only regulates a small number of proteins.^118,119^ The nonenzymatically regulated sites are sensitive to the level of Ac-P in the cell, which vary depending on carbon source.^118^

To gain further insights into factors that trigger lysine acetylation, global acetylome analysis using LFQ was carried out following the growth of *E. coli* in media containing high and low levels of the hexose sugar glucose or the pentose sugar xylose.^121^ It was determined that the specific sugar was not important per se, but the amount of sugar correlated to lysine acetylation patterns. 978 proteins with 3840 lysine-acetylation sites were identified, and 95% of them were identical between glucose and xylose at the same concentration. Conversely, the degree of acetylation at high (4%) glucose concentrations increased by more than 2-fold for 260 acetylation sites on 149 proteins compared to that at low (0.4%) glucose conditions. Similar observations were made for xylose conditions. These observations support the hypothesis that the majority of acetylation in a cell is a direct result of acetate overflow metabolism. Only two enzymes involved in central carbon metabolism had significantly increased levels of acetylation, xylose isomerase (XylA) and phosphoenolpyruvate carboxylase (Ppc). Further examination revealed that acetylation does not regulate the activity of either of these enzymes, suggesting that nonenzymatic acetylation likely targets accessible lysines, rather than specific metabolic pathways. This data support the idea that acetylation occurs from a buildup of metabolic intermediates, under growth conditions that lead to acetate production. This hypothesis raises many interesting questions about how and why bacteria deal with such a phenomenon, and these questions will undoubtedly be answered using MS-based proteomics.

MS-based proteomics can also be used to confirm the involvement or characterize novel enzymes of acetylation. Our lab identified the acetyltransferase YfmK as a novel protein acetylase for the histone-like protein HBsu in *Bacillus subtilis*, using classic epistasis experiments and phenotypic observations.^122^ With purified recombinant proteins, an *in vitro* acetylation assay was performed, and it was demonstrated that HBsu is acetylated in the presence of YfmK and Ac-CoA. MS-based proteomics was used to analyze these *in vitro* reactions to identify specific sites that were acetylated by YfmK. YfmK had a preference to acetylate the C-terminally localized lysine residues in HBsu. In addition, endogenous levels of HBsu acetylation in wild-type and Δ*yfmK* mutant strains were measured using the MS technique, called parallel reaction monitoring. Three acetylation sites were identified, and K80 acetylation was reliably quantified. Acetylation at this site was reduced 15-fold in the *yfmK* mutant, suggesting that this is a bona fide *in vivo* target of YfmK. The complete list of substrates for YfmK are not known, and further characterizations are ongoing.

In *E. coli*, there are 26 genes annotated as GNATs, most of which are of unknown function. To uncover if any of these enzymes are KATs, a strain was built that cannot produce Ac-P and is deleted for *yfiQ*.^123^ In this background, there was still residual protein acetylation, as determined by Western blot using antiacetyllysine antibodies, which suggested that additional KATs exist. Overexpression of each GNAT revealed that four of these putative GNAT proteins functioned as KATs. Mutation of conserved active site residues in these four enzymes, RimI, YiaC, YjaB, and PhnO, abolished the KAT activity. MS-based proteomics analysis identified the substrates for each of these enzymes. YfiQ had the largest number of substrates (364 proteins), followed by YiaC (251), YiaB (128), RimI (11), and PhnO (10). Many enzymes in glycolysis and the ribosomal subunits were acetylated by these enzymes, with some acetylated by more than one KAT. Interestingly, only 29 of the 592 Ac-P-dependent sites^118^ were acetylated by one of these enzymes, which reinforces the enzymatic specificity and distinct functions. Further characterization of the regulation of these KATs will further our understanding of how acetylation influences central metabolism and translation.

## Perspectives

The protein acylation field in bacteria has exploded since 2008. The acetylomes of more than 50 different species are now available, with catalogs of hundreds of proteins, reviewed here and previously.^7−14^ In addition, other acyl modifications are being identified and continually studied in bacteria, including succinylation,^63−65,77,78,80,124−126^ malonylation,^75,127,128^ and propionylation.^129−131^ As we continue to discover more PTMs, the need to understand their physiological relevance becomes greater. It is important to note that among acylome studies there is a large variation in modified sites and proteins that are identified, often with little overlap between studies, even for the same species. One reason for this is that analyses are performed under different growth conditions, including media, i.e., minimal media with different carbon sources or rich media, stress conditions, and growth phase. In addition, each lab may use a different proteomic workflow and different mass spectrometry instrumentation. Finally, the quality of acyl-antibodies for enrichment varies, where some laboratories use mixtures, but others do not. This may greatly influence the downstream results. Although there may be little overlap between studies, building up a catalog of all possible modifications in a species is valuable and can prioritize candidates for further evaluation.

Acylations are challenging to study because of their dynamic nature and relatively low stoichiometry. In addition, with these newer acyl modifications, we do not have many developed tools for study. For example, there are no widely accepted substitution mutations to mimic the modified state as there are for acetylation and succinylation. However, there might be opportunities to chemically modify lysine residues to study some of these other modifications using *in vitro* biochemical approaches. Indeed, recently, chemical mutagenesis techniques have been described to insert PTMs into recombinant proteins, including acetylation and succinylation.^132^ Perhaps, these techniques can be adapted for some of the other acyl modifications as well. Development of more tools to learn about these different acyl modifications is essential if we are to understand if these are more than just nonenzymatic consequences of metabolism or if they are biologically meaningful. In addition, identification and characterization of enzymes that add these groups and remove them is essential. Perhaps, it would be wise to begin with known acetyltransferases and deacetylases to determine if these enzymes have expanded activities. This is already known for some human enzymes, including the sirtuin SIRT5, which has demalonylase and desuccinylase activity,^133^ and SIRT7, which is a deacetylase and desuccinylase.^134^

As top-down MS reagents, protocols, and instrumentation have significantly improved, the opportunity to study the acylation of single intact bacterial proteins may be at an all-time high. Of course, depending on protein size, a middle-down approach may be the best choice to evaluate the PTM landscape. Exploring top-down MS would reveal information about stoichiometry and different proteoforms that exist in the cell, possibly identifying combinations of different acyl modifications for each protein. As mentioned here, there is growing evidence that there might be crosstalk among PTMs in bacteria.^63,77,79,80,83,125^ As of now, this is largely based on the observation that a single lysine residue can be modified by multiple PTMs. Top-down MS would allow for the examination of all combinations of PTMs and different proteoforms that exist in the cell, under different growth conditions, or in the presence of environmental stresses. Quantitative proteomic approaches would allow for the detection of increased abundances of specific proteoforms. Examination of the physiological significance of these PTMs in combination with intact MS data would provide solid support for and an understanding of crosstalk among PTMs. In addition, top-down MS analysis in a strain background deleted for any known acyltransferases or deacylases could provide information about substrate specificity and provide further information about how the PTM landscape is set and changed in the presence of different environmental cues.

There will undoubtedly be more acylomes characterized in the next decade, from previously uncharacterized bacteria, bacteria grown under different stress conditions, or pathogens. So far, most of the pathogenic acylomes that have been characterized were from strains grown under laboratory conditions, where acetylated virulence factors were identified. It will be interesting to evaluate the acetylome or any acylome from pathogens isolated *in vivo* from model organisms. This might be technically challenging to obtain enough starting material for a reliable analysis. Quantitative MS analyses, especially iTRAQ or TMT, may be useful to compare laboratory grown cells and those that were grown *in vivo*. These analyses would provide a clearer picture of the exact role, if any, that protein acylation plays in bacterial virulence.

Many published acylomes identify proteins that are involved in adhesion or quorum sensing, as modified by PTMs. These pathways are involved in cell–cell or cell–host interactions or communications. An interesting extension of acetylomics might be in the large-scale characterization of populations of bacteria, especially the microbiota. This analysis could aid in our understanding of how bacteria interact with one another or the host and if acylation plays a role in such interactions. Zhang et al. was one of the first teams to characterize the acetylome, succinylome, and propionylome of the human gut microbiota.^135,136^ From 6 microbiomes, they identified 60,957 acetylation sites, 20,914 propionylation sites, and 17,089 succinylation sites. As this is a massive data set, an important contribution was the development of a “meta-PTMomics” workflow that involved serial enrichments of PTMs, high resolution MS, and an unrestricted database search. From this work, it was demonstrated that lysine acylations are widespread and may enable communication among the microbiota, especially regarding glycolysis and the production of short-chain fatty acids. The levels of these modifications varied among the different species that had different metabolic properties. With this groundwork established, microbiome analyses should be expanded upon and examined in health and disease. New software platforms should be developed to handle these very large data sets generated from this type of experiment. Characterization of the acylomes of bacterial populations represents an exciting future direction for the field. If the first 15 years of this field are an indication of what’s to come, more groundbreaking discoveries will surely be made in the next decade of bacterial PTM research.

## Abbreviations

PTM: post-translational modification
MS: mass spectrometry
kDa: kilodalton
nESI: nanoelectrospray ionization
CID: collision-induced dissociation
HCD: higher energy collisional dissociation
ECD: electron capture dissociation
ETD: electron transfer dissociation
LFQ: label-free quantification
SILAC: stable isotope labeling of amino acids in cell culture
iTRAQ: isobaric tagging for relative and absolute quantification
TMT: tandem mass tag
*m*/*z*: mass to charge ratio
EMSA: electrophoretic mobility shift assay
USD: United States dollar
LB: lysogeny broth
OD: optical density
MDR: multidrug resistant
VISA: vancomycin intermediate *S. aureus*
EPS: exopolysaccharide
CPS: capsule polysaccharide
KAT: lysine acetyltransferase
KDAC: lysine deacetylase
Ac-P: acetyl-phosphate
Ac-CoA: acetyl-CoA

## References

[ref1] Kitamura N.; Galligan J. J. A global view of the human post-translational modification landscape. Biochem. J. 2023, 480 (16), 1241–1265. 10.1042/BCJ20220251. 37610048 PMC10586784

[ref2] Levene P. A.; Alsberg C. L. The cleavage products of vitellin. J. Biol. Chem. 1906, 2 (1), 127–133. 10.1016/S0021-9258(17)46054-6.

[ref3] Allfrey V. G.; Faulkner R.; Mirsky A. E. Acetylation and methylation of histones and their possible role in the regulation of RNA synthesis. Proc. Nat. Acad. Sci. 1964, 51 (5), 786–794. 10.1073/pnas.51.5.786. 14172992 PMC300163

[ref4] Wolfe A. J.; Conley M. P.; Berg H. C. Acetyladenylate plays a role in controlling the direction of flagellar rotation. Proc. Natl. Acad. Sci. U. S. A. 1988, 85 (18), 6711–6715. 10.1073/pnas.85.18.6711. 2901103 PMC282047

[ref5] Barak R.; Welch M.; Yanovsky A.; Oosawa K.; Eisenbach M. Acetyladenylate or its derivative acetylates the chemotaxis protein CheY in vitro and increases its activity at the flagellar switch. Biochem 1992, 31 (41), 10099–10107. 10.1021/bi00156a033. 1390767

[ref6] Yu B. J.; Kim J. A.; Moon J. H.; Ryu S. E.; Pan J. G. The diversity of lysine-acetylated proteins in *Escherichia coli*. J. Microbiol. Biotechnol. 2008, 18 (9), 1529–1536. 18852508

[ref7] Macek B.; Forchhammer K.; Hardouin J.; Weber-Ban E.; Grangeasse C.; Mijakovic I. Protein post-translational modifications in bacteria. Nat. Rev. Microbiol 2019, 17 (11), 651–664. 10.1038/s41579-019-0243-0. 31485032

[ref8] Ouidir T.; Kentache T.; Hardouin J. Protein lysine acetylation in bacteria: Current state of the art. Proteomics 2016, 16 (2), 301–309. 10.1002/pmic.201500258. 26390373

[ref9] Luu J.; Carabetta V. J. Contribution of N(ε)-lysine acetylation towards regulation of bacterial pathogenesis. mSystems 2021, 6 (4), e0042221 10.1128/mSystems.00422-21. 34427523 PMC8407419

[ref10] VanDrisse C. M.; Escalante-Semerena J. C. Protein acetylation in bacteria. Annu. Rev. Microbiol. 2019, 73, 111–132. 10.1146/annurev-micro-020518-115526. 31091420 PMC6736716

[ref11] Christensen D. G.; Xie X.; Basisty N.; Byrnes J.; McSweeney S.; Schilling B.; Wolfe A. J. Post-translational protein acetylation: an elegant mechanism for bacteria to dynamically regulate metabolic functions. Front Microbiol 2019, 10, 1604 10.3389/fmicb.2019.01604. 31354686 PMC6640162

[ref12] Christensen D. G.; Baumgartner J. T.; Xie X.; Jew K. M.; Basisty N.; Schilling B.; Kuhn M. L.; Wolfe A. J. Mechanisms, detection, and relevance of protein acetylation in prokaryotes. mBio 2019, 10 (2), e02708-18 10.1128/mBio.02708-18. 30967470 PMC6456759

[ref13] Ren J.; Sang Y.; Lu J.; Yao Y. F. Protein acetylation and its role in bacterial virulence. Trends Microbiol 2017, 25 (9), 768–779. 10.1016/j.tim.2017.04.001. 28462789

[ref14] Carabetta V. J.; Cristea I. M. Regulation, function, and detection of protein acetylation in bacteria. J. Bacteriol. 2017, 199 (16), e00107-17 10.1128/JB.00107-17. 28439035 PMC5527388

[ref15] Keenan E. K.; Zachman D. K.; Hirschey M. D. Discovering the landscape of protein modifications. Mol. Cell 2021, 81 (9), 1868–1878. 10.1016/j.molcel.2021.03.015. 33798408 PMC8106652

[ref16] Dupree E. J.; Jayathirtha M.; Yorkey H.; Mihasan M.; Petre B. A.; Darie C. C. A critical review of bottom-up proteomics: The good, the bad, and the future of this field. Proteomes 2020, 8 (3), 14 10.3390/proteomes8030014. 32640657 PMC7564415

[ref17] Moradian A.; Kalli A.; Sweredoski M. J.; Hess S. The top-down, middle-down, and bottom-up mass spectrometry approaches for characterization of histone variants and their post-translational modifications. Proteomics 2014, 14 (4–5), 489–497. 10.1002/pmic.201300256. 24339419

[ref18] Larance M.; Lamond A. I. Multidimensional proteomics for cell biology. Nat. Rev. Mol. Cell Biol. 2015, 16 (5), 269–280. 10.1038/nrm3970. 25857810

[ref19] Catherman A. D.; Skinner O. S.; Kelleher N. L. Top Down proteomics: facts and perspectives. Biochem. Biophys. Res. Commun. 2014, 445 (4), 683–693. 10.1016/j.bbrc.2014.02.041. 24556311 PMC4103433

[ref20] Cannon J.; Lohnes K.; Wynne C.; Wang Y.; Edwards N.; Fenselau C. High-throughput middle-down analysis using an orbitrap. J. Proteome Res. 2010, 9 (8), 3886–3890. 10.1021/pr1000994. 20557100 PMC2917504

[ref21] Pandeswari P. B.; Sabareesh V. Middle-down approach: a choice to sequence and characterize proteins/proteomes by mass spectrometry. RSC Adv. 2019, 9 (1), 313–344. 10.1039/C8RA07200K. 35521579 PMC9059502

[ref22] Young N. L.; DiMaggio P. A.; Plazas-Mayorca M. D.; Baliban R. C.; Floudas C. A.; Garcia B. A. High throughput characterization of combinatorial histone codes. Mol. Cell Proteomics 2009, 8 (10), 2266–2284. 10.1074/mcp.M900238-MCP200. 19654425 PMC2758755

[ref23] Kalli A.; Sweredoski M. J.; Hess S. Data-dependent middle-down nano-liquid chromatography-electron capture dissociation-tandem mass spectrometry: an application for the analysis of unfractionated histones. Anal. Chem. 2013, 85 (7), 3501–3507. 10.1021/ac303103b. 23448339

[ref24] Schachner L. F.; Ives A. N.; McGee J. P.; Melani R. D.; Kafader J. O.; Compton P. D.; Patrie S. M.; Kelleher N. L. Standard proteoforms and their complexes for native mass spectrometry. J. Am. Soc. Mass Spectrom. 2019, 30 (7), 1190–1198. 10.1007/s13361-019-02191-w. 30963455 PMC6592724

[ref25] Chen B.; Peng Y.; Valeja S. G.; Xiu L.; Alpert A. J.; Ge Y. Online hydrophobic interaction chromatography–mass spectrometry for top-down proteomics. Anal. Chem. 2016, 88 (3), 1885–1891. 10.1021/acs.analchem.5b04285. 26729044 PMC4947469

[ref26] Xiu L.; Valeja S. G.; Alpert A. J.; Jin S.; Ge Y. Effective protein separation by coupling hydrophobic interaction and reverse phase chromatography for top-down proteomics. Anal. Chem. 2014, 86 (15), 7899–7906. 10.1021/ac501836k. 24968279 PMC4144745

[ref27] Donnelly D. P.; Rawlins C. M.; DeHart C. J.; Fornelli L.; Schachner L. F.; Lin Z.; Lippens J. L.; Aluri K. C.; Sarin R.; Chen B.; et al. Best practices and benchmarks for intact protein analysis for top-down mass spectrometry. Nat. Meth 2019, 16 (7), 587–594. 10.1038/s41592-019-0457-0. PMC6719561 31249407

[ref28] Schaffer L. V.; Millikin R. J.; Miller R. M.; Anderson L. C.; Fellers R. T.; Ge Y.; Kelleher N. L.; LeDuc R. D.; Liu X.; Payne S. H.; et al. Identification and quantification of proteoforms by mass spectrometry. Proteomics 2019, 19 (10), e1800361 10.1002/pmic.201800361. 31050378 PMC6602557

[ref29] Chait B. T. Mass spectrometry: Bottom-up or top-down?. Science 2006, 314 (5796), 65–66. 10.1126/science.1133987. 17023639

[ref30] Meyer B.; Papasotiriou D. G.; Karas M. 100% protein sequence coverage: a modern form of surrealism in proteomics. Amino Acids 2011, 41 (2), 291–310. 10.1007/s00726-010-0680-6. 20625782

[ref31] Brandi J.; Noberini R.; Bonaldi T.; Cecconi D. Advances in enrichment methods for mass spectrometry-based proteomics analysis of post-translational modifications. J. Chromat A 2022, 1678, 463352 10.1016/j.chroma.2022.463352. 35896048

[ref32] Greco T. M.; Miteva Y.; Conlon F. L.; Cristea I. M. Complementary proteomic analysis of protein complexes. Methods Mol. Biol. 2012, 917, 391–407. 10.1007/978-1-61779-992-1_22. 22956100 PMC3616387

[ref33] Gundry R. L.; White M. Y.; Murray C. I.; Kane L. A.; Fu Q.; Stanley B. A.; Van Eyk J. E. Preparation of proteins and peptides for mass spectrometry analysis in a bottom-up proteomics workflow. Curr. Protoc Mol. Biol. 2010, 90, 1 10.1002/0471142727.mb1025s88. PMC2905857 19816929

[ref34] Wiśniewski J. R. Filter aided sample preparation - A tutorial. Anal. Chim. Acta 2019, 1090, 23–30. 10.1016/j.aca.2019.08.032. 31655642

[ref35] Scigelova M.; Hornshaw M.; Giannakopulos A.; Makarov A. Fourier transform mass spectrometry. Mol. Cell Proteomics 2011, 10 (7), M111.009431 10.1074/mcp.M111.009431. PMC3134075 21742802

[ref36] Doll S.; Burlingame A. L. Mass spectrometry-based detection and assignment of protein posttranslational modifications. ACS Chem. Biol. 2015, 10 (1), 63–71. 10.1021/cb500904b. 25541750 PMC4301092

[ref37] Wu Z.; Roberts D. S.; Melby J. A.; Wenger K.; Wetzel M.; Gu Y.; Ramanathan S. G.; Bayne E. F.; Liu X.; Sun R.; et al. MASH Explorer: A Universal software environment for top-down proteomics. J. Proteome Res. 2020, 19 (9), 3867–3876. 10.1021/acs.jproteome.0c00469. 32786689 PMC7728713

[ref38] Cai W.; Guner H.; Gregorich Z. R.; Chen A. J.; Ayaz-Guner S.; Peng Y.; Valeja S. G.; Liu X.; Ge Y. MASH Suite Pro: A comprehensive software tool for top-down proteomics. Mol. Cell Proteomics 2016, 15 (2), 703–714. 10.1074/mcp.O115.054387. 26598644 PMC4739683

[ref39] Guner H.; Close P. L.; Cai W.; Zhang H.; Peng Y.; Gregorich Z. R.; Ge Y. MASH Suite: a user-friendly and versatile software interface for high-resolution mass spectrometry data interpretation and visualization. J. Am. Soc. Mass Spectrom. 2014, 25 (3), 464–470. 10.1007/s13361-013-0789-4. 24385400 PMC3940544

[ref40] Solntsev S. K.; Shortreed M. R.; Frey B. L.; Smith L. M. Enhanced global post-translational modification discovery with MetaMorpheus. J. Proteome Res. 2018, 17 (5), 1844–1851. 10.1021/acs.jproteome.7b00873. 29578715

[ref41] Park J.; Piehowski P. D.; Wilkins C.; Zhou M.; Mendoza J.; Fujimoto G. M.; Gibbons B. C.; Shaw J. B.; Shen Y.; Shukla A. K.; et al. Informed-Proteomics: open-source software package for top-down proteomics. Nat. Meth 2017, 14 (9), 909–914. 10.1038/nmeth.4388. PMC5578875 28783154

[ref42] Cesnik A. J.; Shortreed M. R.; Schaffer L. V.; Knoener R. A.; Frey B. L.; Scalf M.; Solntsev S. K.; Dai Y.; Gasch A. P.; Smith L. M. Proteoform Suite: Software for constructing, quantifying, and visualizing proteoform families. J. Proteome Res. 2018, 17 (1), 568–578. 10.1021/acs.jproteome.7b00685. 29195273 PMC5770237

[ref43] Schaffer L. V.; Shortreed M. R.; Cesnik A. J.; Frey B. L.; Solntsev S. K.; Scalf M.; Smith L. M. Expanding proteoform identifications in top-down proteomic analyses by constructing proteoform families. Anal. Chem. 2018, 90 (2), 1325–1333. 10.1021/acs.analchem.7b04221. 29227670 PMC5807004

[ref44] Kou Q.; Wu S.; Tolić N.; Paša-Tolić L.; Liu Y.; Liu X. A mass graph-based approach for the identification of modified proteoforms using top-down tandem mass spectra. Bioinformatics 2017, 33 (9), 1309–1316. 10.1093/bioinformatics/btw806. 28453668 PMC5860502

[ref45] Kou Q.; Xun L.; Liu X. TopPIC: a software tool for top-down mass spectrometry-based proteoform identification and characterization. Bioinformatics 2016, 32 (22), 3495–3497. 10.1093/bioinformatics/btw398. 27423895 PMC5181555

[ref46] Zhu W.; Smith J. W.; Huang C. M. Mass spectrometry-based label-free quantitative proteomics. J. Biomed Biotechnol 2010, 2010, 840518 10.1155/2010/840518. 19911078 PMC2775274

[ref47] Ong S. E.; Blagoev B.; Kratchmarova I.; Kristensen D. B.; Steen H.; Pandey A.; Mann M. Stable isotope labeling by amino acids in cell culture, SILAC, as a simple and accurate approach to expression proteomics. Mol. Cell Proteomics 2002, 1 (5), 376–386. 10.1074/mcp.M200025-MCP200. 12118079

[ref48] Ong S. E.; Mann M. A practical recipe for stable isotope labeling by amino acids in cell culture (SILAC). Nat. Protoc 2006, 1 (6), 2650–2660. 10.1038/nprot.2006.427. 17406521

[ref49] Zhang G.; Neubert T. A. Use of stable isotope labeling by amino acids in cell culture (SILAC) for phosphotyrosine protein identification and quantitation. Methods Mol. Biol. 2009, 527, 79–92. 10.1007/978-1-60327-834-8_7. 19241007 PMC3757925

[ref50] Wiese S.; Reidegeld K. A.; Meyer H. E.; Warscheid B. Protein labeling by iTRAQ: A new tool for quantitative mass spectrometry in proteome research. Proteomics 2007, 7 (3), 340–350. 10.1002/pmic.200600422. 17177251

[ref51] Thompson A.; Schafer J.; Kuhn K.; Kienle S.; Schwarz J.; Schmidt G.; Neumann T.; Hamon C. Tandem mass tags: a novel quantification strategy for comparative analysis of complex protein mixtures by MS/MS. Anal. Chem. 2003, 75 (8), 1895–1904. 10.1021/ac0262560. 12713048

[ref52] Zecha J.; Satpathy S.; Kanashova T.; Avanessian S. C.; Kane M. H.; Clauser K. R.; Mertins P.; Carr S. A.; Kuster B. TMT labeling for the masses: A robust and cost-efficient, in-solution labeling approach. Mol. Cell Proteomics 2019, 18 (7), 1468–1478. 10.1074/mcp.TIR119.001385. 30967486 PMC6601210

[ref53] Wang Y.; Wang F.; Bao X.; Fu L. Systematic analysis of lysine acetylome reveals potential functions of lysine acetylation in *Shewanella baltica*, the specific spoilage organism of aquatic products. J. Proteomics 2019, 205, 103419 10.1016/j.jprot.2019.103419. 31212084

[ref54] Yoshida A.; Yoshida M.; Kuzuyama T.; Nishiyama M.; Kosono S. Protein acetylation on 2-isopropylmalate synthase from *Thermus thermophilus* HB27. Extremophiles 2019, 23 (4), 377–388. 10.1007/s00792-019-01090-y. 30919057

[ref55] Wang L.; Li X.; Wang M.; Ma X.; Song F.; Hu J.; Liang W.; Liang W. Carbon metabolism and the ROS scavenging system participate in *Nostoc flagelliforme*’s adaptive response to dehydration conditions through protein acetylation. J. Proteome Res. 2022, 21 (2), 482–493. 10.1021/acs.jproteome.1c00823. 35020403

[ref56] Greiner-Haas F.; Bergen M. v.; Sawers G.; Lechner U.; Türkowsky D. Changes of the proteome and acetylome during transition into the stationary phase in the organohalide-respiring *Dehalococcoides mccartyi* strain CBDB1. Microorganisms 2021, 9 (2), 365 10.3390/microorganisms9020365. 33673241 PMC7918482

[ref57] Brüser T.; Sanders C. An alternative model of the twin arginine translocation system. Microbiol Res. 2003, 158 (1), 7–17. 10.1078/0944-5013-00176. 12608575

[ref58] Hamsanathan S.; Musser S. M. The Tat protein transport system: intriguing questions and conundrums. FEMS Microbiol Lett. 2018, 365 (12), fny123 10.1093/femsle/fny123. 29897510 PMC5995166

[ref59] Simone D.; Bay D. C.; Leach T.; Turner R. J. Diversity and evolution of bacterial twin arginine translocase protein, TatC, reveals a protein secretion system that is evolving to fit its environmental niche. PLoS One 2013, 8 (11), e78742 10.1371/journal.pone.0078742. 24236045 PMC3827258

[ref60] Liu Y.; Liu X.; Dong X.; Yin Z.; Xie Z.; Luo Y. Systematic analysis of lysine acetylation reveals diverse functions in *Azorhizobium caulinodans* strain ORS571. Microbiol Spect 2023, 11 (1), e03539-22 10.1128/spectrum.03539-22. PMC9927263 36475778

[ref61] Ito H.; Watanabe H.; Takehisa M.; Iizuka H. Isolation and identification of radiation-resistant cocci belonging to the genus Deinococcus from sewage sludges and animal feeds. Agric. Biol. Chem. 1983, 47 (6), 1239–1247. 10.1080/00021369.1983.10866087.

[ref62] Zhang Y.; Li N.; Wei Q.; Min R.; Liu F.; Wang F.; Deng Y. Lysine acetylome profiling reveals diverse functions of acetylation in *Deinococcus radiodurans*. Microbiol Spectr 2022, 10 (5), e0101621 10.1128/spectrum.01016-21. 35972276 PMC9603093

[ref63] Wu L.; Gong T.; Zhou X.; Zeng J.; Huang R.; Wu Y.; Li Y. Global analysis of lysine succinylome in the periodontal pathogen *Porphyromonas gingivalis*. Mol. Oral Microbiol 2019, 34 (2), 74–83. 10.1111/omi.12255. 30672658

[ref64] Zhou C.; Dai J.; Lu H.; Chen Z.; Guo M.; He Y.; Gao K.; Ge T.; Jin J.; Wang L.; et al. Succinylome analysis reveals the involvement of lysine succinylation in the extreme resistance of *Deinococcus radiodurans*. Proteomics 2019, 19 (20), 1900158 10.1002/pmic.201900158. 31487437

[ref65] Zhao Y.; Han Y.; Sun Y.; Wei Z.; Chen J.; Niu X.; An Q.; Zhang L.; Qi R.; Gao X. Comprehensive succinylome profiling reveals the pivotal role of lysine succinylation in energy metabolism and quorum sensing of *Staphylococcus epidermidis*. Front Microbiol 2021, 11, 632367 10.3389/fmicb.2020.632367. 33597936 PMC7882547

[ref66] Weinert B. T.; Schölz C.; Wagner S. A.; Iesmantavicius V.; Su D.; Daniel J. A.; Choudhary C. Lysine succinylation is a frequently occurring modification in prokaryotes and eukaryotes and extensively overlaps with acetylation. Cell Rep 2013, 4 (4), 842–851. 10.1016/j.celrep.2013.07.024. 23954790

[ref67] Pan J.; Chen R.; Li C.; Li W.; Ye Z. Global analysis of protein lysine succinylation profiles and their overlap with lysine acetylation in the marine bacterium *Vibrio parahemolyticus*. J. Proteome Res. 2015, 14 (10), 4309–4318. 10.1021/acs.jproteome.5b00485. 26369940

[ref68] Colak G.; Xie Z.; Zhu A. Y.; Dai L.; Lu Z.; Zhang Y.; Wan X.; Chen Y.; Cha Y. H.; Lin H.; et al. Identification of lysine succinylation substrates and the succinylation regulatory enzyme CobB in *Escherichia coli*. Mol. Cell Proteomics 2013, 12 (12), 3509–3520. 10.1074/mcp.M113.031567. 24176774 PMC3861704

[ref69] Zhang M.; Liu T.; Wang L.; Huang Y.; Fan R.; Ma K.; Kan Y.; Tan M.; Xu J.-Y. Global landscape of lysine acylomes in *Bacillus subtilis*. J. Proteomics 2023, 271, 104767 10.1016/j.jprot.2022.104767. 36336260

[ref70] Leutert M.; Entwisle S. W.; Villén J. Decoding post-translational modification crosstalk with proteomics. Mol. Cell Proteomics 2021, 20, 100129 10.1016/j.mcpro.2021.100129. 34339852 PMC8430371

[ref71] Ravikumar V.; Nalpas N. C.; Anselm V.; Krug K.; Lenuzzi M.; Šestak M. S.; Domazet-Lošo T.; Mijakovic I.; Macek B. In-depth analysis of *Bacillus subtilis* proteome identifies new ORFs and traces the evolutionary history of modified proteins. Sci. Rep 2018, 8 (1), 17246 10.1038/s41598-018-35589-9. 30467398 PMC6250715

[ref72] Boersema P. J.; Raijmakers R.; Lemeer S.; Mohammed S.; Heck A. J. Multiplex peptide stable isotope dimethyl labeling for quantitative proteomics. Nat. Protoc 2009, 4 (4), 484–494. 10.1038/nprot.2009.21. 19300442

[ref73] Xu J. Y.; Xu Z.; Liu X.; Tan M.; Ye B. C. Protein acetylation and butyrylation regulate the phenotype and metabolic shifts of the endospore-forming *Clostridium acetobutylicum*. Mol. Cell Proteomics 2018, 17 (6), 1156–1169. 10.1074/mcp.RA117.000372. 29523768 PMC5986239

[ref74] Zhang X.; Chen J.; Dong Q.; Zhu J.; Peng R.; He C.; Li Y.; Lin R.; Jiang P.; Zheng M.; et al. Lysine acylation modification landscape of *Brucella abortus* proteome and its virulent proteins. Front Cell Develop Biol. 2022, 10, 839822 10.3389/fcell.2022.839822. PMC8921143 35300419

[ref75] Shi Y.; Zhu J.; Xu Y.; Tang X.; Yang Z.; Huang A. Malonyl-proteome profiles of *Staphylococcus aureus* reveal lysine malonylation modification in enzymes involved in energy metabolism. Proteome Sci. 2021, 19 (1), 1 10.1186/s12953-020-00169-1. 33436009 PMC7802289

[ref76] Yang Y.; Zhang H.; Guo Z.; Zou S.; Long F.; Wu J.; Li P.; Zhao G. P.; Zhao W. Global insights into lysine acylomes reveal crosstalk between lysine acetylation and succinylation in *Streptomyces coelicolor* metabolic pathways. Mol. Cell Proteomics 2021, 20, 100148 10.1016/j.mcpro.2021.100148. 34530157 PMC8498004

[ref77] Tan L.; Yang Y.; Shang W.; Hu Z.; Peng H.; Li S.; Hu X.; Rao X. Identification of lysine succinylome and acetylome in the vancomycin-intermediate *Staphylococcus aureus* XN108. Microbiol Spectr 2022, 10 (6), e03481-22 10.1128/spectrum.03481-22. 36374118 PMC9769639

[ref78] Fu Y.; Zhang L.; Song H.; Liao J.; Lin L.; Jiang W.; Wu X.; Wang G. Acetylome and succinylome profiling of *Edwardsiella tarda* reveals key roles of both lysine acylations in bacterial antibiotic resistance. Antibiotics 2022, 11 (7), 841 10.3390/antibiotics11070841. 35884095 PMC9312108

[ref79] Sun L.; Yao Z.; Guo Z.; Zhang L.; Wang Y.; Mao R.; Lin Y.; Fu Y.; Lin X. Comprehensive analysis of the lysine acetylome in *Aeromonas hydrophila* reveals cross-talk between lysine acetylation and succinylation in LuxS. Emerg Microbes Infect 2019, 8 (1), 1229–1239. 10.1080/22221751.2019.1656549. 31448697 PMC6735345

[ref80] Zeng F.; Pang H.; Chen Y.; Zheng H.; Li W.; Ramanathan S.; Hoare R.; Monaghan S. J.; Lin X.; Jian J. First succinylome profiling of *Vibrio alginolyticus* reveals key role of lysine succinylation in cellular metabolism and virulence. Front Cell Infect Microbiol 2021, 10, 626574 10.3389/fcimb.2020.626574. 33614530 PMC7892601

[ref81] Zeng J.; Wu L.; Chen Q.; Wang L.; Qiu W.; Zheng X.; Yin X.; Liu J.; Ren Y.; Li Y. Comprehensive profiling of protein lysine acetylation and its overlap with lysine succinylation in the *Porphyromonas gingivalis* fimbriated strain ATCC 33277. Mol. Oral Microbiol 2020, 35 (6), 240–250. 10.1111/omi.12312. 32939976

[ref82] Sun L.; Yao Z.; Guo Z.; Zhang L.; Wang Y.; Mao R.; Lin Y.; Fu Y.; Lin X. Comprehensive analysis of the lysine acetylome in *Aeromonas hydrophila* reveals cross-talk between lysine acetylation and succinylation in LuxS. Emerg Microbes Infect 2019, 8 (1), 1229–1239. 10.1080/22221751.2019.1656549. 31448697 PMC6735345

[ref83] Yao Z.; Guo Z.; Wang Y.; Li W.; Fu Y.; Lin Y.; Lin W.; Lin X. Integrated succinylome and metabolome profiling reveals crucial role of S-ribosylhomocysteine lyase in quorum sensing and metabolism of *Aeromonas hydrophila*. Mol. Cell Proteomics 2019, 18 (2), 200–215. 10.1074/mcp.RA118.001035. 30352804 PMC6356075

[ref84] Sato M.; Yoshida Y.; Nagano K.; Hasegawa Y.; Takebe J.; Yoshimura F. Three CoA transferases involved in the production of short chain fatty acids in *Porphyromonas gingivalis*. Front Microbiol 2016, 7, 1146 10.3389/fmicb.2016.01146. 27486457 PMC4949257

[ref85] Takahashi N.; Sato T.; Yamada T. Metabolic pathways for cytotoxic end product formation from glutamate- and aspartate-containing peptides by *Porphyromonas gingivalis*. J. Bacteriol. 2000, 182 (17), 4704–4710. 10.1128/JB.182.17.4704-4710.2000. 10940008 PMC111344

[ref86] Wu L.; Gong T.; Zhou X.; Zeng J.; Huang R.; Wu Y.; Li Y. Global analysis of lysine succinylome in the periodontal pathogen *Porphyromonas gingivalis*. Mol. Oral Microbiol 2019, 34 (2), 74–83. 10.1111/omi.12255. 30672658

[ref87] Khatoon Z.; McTiernan C. D.; Suuronen E. J.; Mah T. F.; Alarcon E. I. Bacterial biofilm formation on implantable devices and approaches to its treatment and prevention. Heliyon 2018, 4 (12), e01067 10.1016/j.heliyon.2018.e01067. 30619958 PMC6312881

[ref88] Roilides E.; Simitsopoulou M.; Katragkou A.; Walsh T. J. How biofilms evade host defenses. Microbiol Spectr 2015, 3 (3), 1 10.1128/microbiolspec.MB-0012-2014. 26185085

[ref89] Adams J. L.; McLean R. J. C. Impact of *rpoS* deletion on *Escherichia coli* biofilms. App EnvironMicrobiol 1999, 65 (9), 4285 10.1128/AEM.65.9.4285-4287.1999. PMC99780 10473455

[ref90] Reverdy A.; Chen Y.; Hunter E.; Gozzi K.; Chai Y. Protein lysine acetylation plays a regulatory role in *Bacillus subtilis* multicellularity. PLoS One 2018, 13 (9), e0204687 10.1371/journal.pone.0204687. 30265683 PMC6161898

[ref91] Lei L.; Zeng J.; Wang L.; Gong T.; Zheng X.; Qiu W.; Zhang R.; Yun L.; Yang Y.; Li Y. Quantitative acetylome analysis reveals involvement of glucosyltransferase acetylation in *Streptococcus mutans* biofilm formation. Environ. Microbiol Rep 2021, 13 (2), 86–97. 10.1111/1758-2229.12907. 33185947

[ref92] Liu M.; Guo L.; Fu Y.; Huo M.; Qi Q.; Zhao G. Bacterial protein acetylation and its role in cellular physiology and metabolic regulation. Biotech Adv. 2021, 53, 107842 10.1016/j.biotechadv.2021.107842. 34624455

[ref93] Schastnaya E.; Doubleday P. F.; Maurer L.; Sauer U. Non-enzymatic acetylation inhibits glycolytic enzymes in i. Cell Rep 2023, 42 (1), 111950 10.1016/j.celrep.2022.111950. 36640332

[ref94] Novak J.; Fabrik I.; Jurnecka D.; Holubova J.; Stanek O.; Sebo P. *Bordetella pertussis* acetylome is shaped by lysine deacetylase Bkd1. J. Proteome Res. 2020, 19 (9), 3680–3696. 10.1021/acs.jproteome.0c00178. 32674575

[ref95] Gaviard C.; Broutin I.; Cosette P.; Dé E.; Jouenne T.; Hardouin J. Lysine succinylation and acetylation in *Pseudomonas aeruginosa*. J. Proteome Res. 2018, 17 (7), 2449–2459. 10.1021/acs.jproteome.8b00210. 29770699

[ref96] Gaviard C.; Cosette P.; Jouenne T.; Hardouin J. LasB and CbpD virulence factors of *Pseudomonas aeruginosa* carry multiple post-translational modifications on their lysine residues. J. Proteome Res. 2019, 18 (3), 923–933. 10.1021/acs.jproteome.8b00556. 30672296

[ref97] Kunz Coyne A. J.; El Ghali A.; Holger D.; Rebold N.; Rybak M. J. Therapeutic strategies for emerging multidrug-resistant *Pseudomonas aeruginosa*. Infect Dis Ther 2022, 11 (2), 661–682. 10.1007/s40121-022-00591-2. 35150435 PMC8960490

[ref98] Tan Y.; Liu W.; Chen Y.; Zhou Y.; Song K.; Cao S.; Zhang Y.; Song Y.; Deng H.; Yang R.; et al. Comparative lysine acetylome analysis of *Y. pestis* YfiQ/CobB mutants reveals that acetylation of SlyA lys73 significantly promotes biofilm formation of *Y. pestis*. Microbiol Spectr 2023, 11 (4), e0046023 10.1128/spectrum.00460-23. 37458592 PMC10433856

[ref99] Jers C.; Ravikumar V.; Lezyk M.; Sultan A.; Sjoling A.; Wai S. N.; Mijakovic I. The global acetylome of the human pathogen *Vibrio cholerae* V52 reveals lysine acetylation of major transcriptional regulators. Front Cell Infect Microbiol 2018, 7, 537 10.3389/fcimb.2017.00537. 29376036 PMC5768985

[ref100] Pang R.; Li Y.; Liao K.; Guo P.; Li Y.; Yang X.; Zhang S.; Lei T.; Wang J.; Chen M.; et al. Genome- and proteome-wide analysis of lysine acetylation in *Vibrio vulnificus* Vv180806 reveals its regulatory roles in virulence and antibiotic resistance. Front Microbiol 2020, 11, 591287 10.3389/fmicb.2020.591287. 33250879 PMC7674927

[ref101] Pang H.; Li W.; Zhang W.; Zhou S.; Hoare R.; Monaghan S. J.; Jian J.; Lin X. Acetylome profiling of *Vibrio alginolyticus* reveals its role in bacterial virulence. J. Proteomics 2020, 211, 103543 10.1016/j.jprot.2019.103543. 31669173

[ref102] Xiao X.; Li W.; Pan Y.; Wang J.; Wei Z.; Wang S.; Wang N.; Jian J.; Pang H. Holistic analysis of lysine acetylation in aquaculture pathogenic bacteria *Vibrio alginolyticus* under bile salt stress. Front Vet Sci. 2023, 10, 1099255 10.3389/fvets.2023.1099255. 37180076 PMC10172577

[ref103] Wang J.; Pang H.; Yin L.; Zeng F.; Wang N.; Hoare R.; Monaghan S. J.; Li W.; Jian J. A comprehensive analysis of the lysine acetylome in the aquatic animals pathogenic bacterium *Vibrio mimicus*. Front Microbiol 2022, 13, 816968 10.3389/fmicb.2022.816968. 35250932 PMC8891801

[ref104] Pan J.; Ye Z.; Cheng Z.; Peng X.; Wen L.; Zhao F. Systematic analysis of the lysine acetylome in *Vibrio parahemolyticus*. J. Proteome Res. 2014, 13 (7), 3294–3302. 10.1021/pr500133t. 24874924

[ref105] Jers C.; Ravikumar V.; Lezyk M.; Sultan A.; Sjöling Å.; Wai S. N.; Mijakovic I. The global acetylome of the human pathogen *Vibrio cholerae* V52 reveals lysine acetylation of major transcriptional regulators. Front Cell Infect Microbiol 2018, 7, 537 10.3389/fcimb.2017.00537. 29376036 PMC5768985

[ref106] Liu Y.-T.; Pan Y.; Lai F.; Yin X.-f.; Ge R.; He Q.-Y.; Sun X. Comprehensive analysis of the lysine acetylome and its potential regulatory roles in the virulence of *Streptococcus pneumoniae*. J. Proteomics 2018, 176, 46–55. 10.1016/j.jprot.2018.01.014. 29386122

[ref107] Yother J. Capsules of *Streptococcus pneumoniae* and other bacteria: Paradigms for polysaccharide biosynthesis and regulation. An Rev. Microbiol 2011, 65 (1), 563–581. 10.1146/annurev.micro.62.081307.162944. 21721938

[ref108] Li L.; Wang W.; Zhang R.; Xu J.; Wang R.; Wang L.; Zhao X.; Li J. First acetyl-proteome profiling of *Salmonella* Typhimurium revealed involvement of lysine acetylation in drug resistance. Vet. Microbiol. 2018, 226, 1–8. 10.1016/j.vetmic.2018.09.024. 30389038

[ref109] Marakasova E.; Ii A.; Nelson K. T.; van Hoek M. L. Proteome wide profiling of N-ε-lysine acetylation reveals a novel mechanism of regulation of the Chitinase activity in *Francisella novicida*. J. Proteome Res. 2020, 19 (4), 1409–1422. 10.1021/acs.jproteome.9b00512. 32056440

[ref110] Chung M. C.; Dean S.; Marakasova E. S.; Nwabueze A. O.; van Hoek M. L. Chitinases are negative regulators of *Francisella novicida* biofilms. PLoS One 2014, 9 (3), e93119 10.1371/journal.pone.0093119. 24664176 PMC3963990

[ref111] Wolfe A. J. Bacterial protein acetylation: new discoveries unanswered questions. Curr. Genet 2016, 62 (2), 335–341. 10.1007/s00294-015-0552-4. 26660885 PMC4826803

[ref112] Lammers M. Post-translational lysine ac(et)ylation in bacteria: A biochemical, structural, and synthetic biological perspective. Front Microbiol 2021, 12, 757179 10.3389/fmicb.2021.757179. 34721364 PMC8556138

[ref113] Hentchel K. L.; Escalante-Semerena J. C. Acylation of biomolecules in prokaryotes: a widespread strategy for the control of biological function and metabolic stress. Microbiol Mol. Biol. Rev. 2015, 79 (3), 321–346. 10.1128/MMBR.00020-15. 26179745 PMC4503791

[ref114] Thao S.; Escalante-Semerena J. C. Control of protein function by reversible Nε-lysine acetylation in bacteria. Curr. Opin Microbiol 2011, 14 (2), 200–204. 10.1016/j.mib.2010.12.013. 21239213 PMC3078959

[ref115] Starai V. J.; Celic I.; Cole R. N.; Boeke J. D.; Escalante-Semerena J. C. Sir2-dependent activation of acetyl-CoA synthetase by deacetylation of active lysine. Science 2002, 298 (5602), 2390–2392. 10.1126/science.1077650. 12493915

[ref116] Starai V. J.; Escalante-Semerena J. C. Identification of the protein acetyltransferase (Pat) enzyme that acetylates acetyl-CoA synthetase in *Salmonella enterica*. J. Mol. Biol. 2004, 340 (5), 1005–1012. 10.1016/j.jmb.2004.05.010. 15236963

[ref117] Starai V. J.; Escalante-Semerena J. C. Acetyl-coenzyme A synthetase (AMP forming). Cell. Mol. Life Sci. 2004, 61 (16), 2020–2030. 10.1007/s00018-004-3448-x. 15316652 PMC11138584

[ref118] Kuhn M. L.; Zemaitaitis B.; Hu L. I.; Sahu A.; Sorensen D.; Minasov G.; Lima B. P.; Scholle M.; Mrksich M.; Anderson W. F.; et al. Structural, kinetic and proteomic characterization of acetyl phosphate-dependent bacterial protein acetylation. PLoS One 2014, 9 (4), e94816 10.1371/journal.pone.0094816. 24756028 PMC3995681

[ref119] Weinert B. T.; Iesmantavicius V.; Wagner S. A.; Schölz C.; Gummesson B.; Beli P.; Nyström T.; Choudhary C. Acetyl-phosphate is a critical determinant of lysine acetylation in *E. coli*. Mol. Cell 2013, 51 (2), 265–272. 10.1016/j.molcel.2013.06.003. 23830618

[ref120] AbouElfetouh A.; Kuhn M. L.; Hu L. I.; Scholle M. D.; Sorensen D. J.; Sahu A. K.; Becher D.; Antelmann H.; Mrksich M.; Anderson W. F.; et al. The *E. coli* sirtuin CobB shows no preference for enzymatic and nonenzymatic lysine acetylation substrate sites. Microbiol Open 2015, 4 (1), 66–83. 10.1002/mbo3.223. PMC4335977 25417765

[ref121] Schilling B.; Basisty N.; Christensen D. G.; Sorensen D.; Orr J. S.; Wolfe A. J.; Rao C. V. Global lysine acetylation in *Escherichia coli* results from growth conditions that favor acetate fermentation. J. Bacteriol. 2019, 201 (9), 1 10.1128/JB.00768-18. PMC6456854 30782634

[ref122] Carabetta V. J.; Greco T. M.; Cristea I. M.; Dubnau D. YfmK is an N(epsilon)-lysine acetyltransferase that directly acetylates the histone-like protein HBsu in *Bacillus subtilis*. Proc. Natl. Acad. Sci. U. S. A. 2019, 116 (9), 3752–3757. 10.1073/pnas.1815511116. 30808761 PMC6397556

[ref123] Christensen D. G.; Meyer J. G.; Baumgartner J. T.; D’Souza A. K.; Nelson W. C.; Payne S. H.; Kuhn M. L.; Schilling B.; Wolfe A. J. Identification of novel protein lysine acetyltransferases in *Escherichia coli*. mBio 2018, 9 (5), 1 10.1128/mBio.01905-18. PMC6199490 30352934

[ref124] Okanishi H.; Kim K.; Fukui K.; Yano T.; Kuramitsu S.; Masui R. Proteome-wide identification of lysine succinylation in thermophilic and mesophilic bacteria. Biochim. Biophys. Acta 2017, 1865 (2), 232–242. 10.1016/j.bbapap.2016.11.009. 27888076

[ref125] Kosono S.; Tamura M.; Suzuki S.; Kawamura Y.; Yoshida A.; Nishiyama M.; Yoshida M. Changes in the acetylome and succinylome of *Bacillus subtilis* in response to carbon source. PLoS One 2015, 10 (6), e0131169 10.1371/journal.pone.0131169. 26098117 PMC4476798

[ref126] Yang M.; Wang Y.; Chen Y.; Cheng Z.; Gu J.; Deng J.; Bi L.; Chen C.; Mo R.; Wang X.; et al. Succinylome analysis reveals the involvement of lysine succinylation in metabolism in pathogenic *Mycobacterium tuberculosis*. Mol. Cell Proteomics 2015, 14 (4), 796–811. 10.1074/mcp.M114.045922. 25605462 PMC4390261

[ref127] Qian L.; Nie L.; Chen M.; Liu P.; Zhu J.; Zhai L.; Tao S.-c.; Cheng Z.; Zhao Y.; Tan M. Global profiling of protein lysine malonylation in *Escherichia coli* reveals its role in energy metabolism. J. Proteome Res. 2016, 15 (6), 2060–2071. 10.1021/acs.jproteome.6b00264. 27183143

[ref128] Fan B.; Li Y.-L.; Li L.; Peng X.-J.; Bu C.; Wu X.-Q.; Borriss R. Malonylome analysis of rhizobacterium *Bacillus amyloliquefaciens* FZB42 reveals involvement of lysine malonylation in polyketide synthesis and plant-bacteria interactions. J. Proteomics 2017, 154, 1–12. 10.1016/j.jprot.2016.11.022. 27939684

[ref129] Okanishi H.; Kim K.; Masui R.; Kuramitsu S. Proteome-wide identification of lysine propionylation in thermophilic and mesophilic bacteria: *Geobacillus kaustophilus*, *Thermus thermophilus*, *Escherichia coli*, *Bacillus subtilis*, and *Rhodothermus marinus*. Extremophiles 2017, 21 (2), 283–296. 10.1007/s00792-016-0901-3. 27928680

[ref130] Sun M.; Xu J.; Wu Z.; Zhai L.; Liu C.; Cheng Z.; Xu G.; Tao S.; Ye B.-C.; Zhao Y.; et al. Characterization of protein lysine propionylation in *Escherichia coli*: Global profiling, dynamic change, and enzymatic regulation. J. Proteome Res. 2016, 15 (12), 4696–4708. 10.1021/acs.jproteome.6b00798. 27804304

[ref131] Okanishi H.; Kim K.; Masui R.; Kuramitsu S. Lysine propionylation is a prevalent post-translational modification in *Thermus thermophilus*. Mol. Cell Proteomics 2014, 13 (9), 2382–2398. 10.1074/mcp.M113.035659. 24938286 PMC4159656

[ref132] Harel O.; Jbara M. Posttranslational chemical mutagenesis methods to insert posttranslational modifications into recombinant proteins. Molecules 2022, 27 (14), 4389 10.3390/molecules27144389. 35889261 PMC9316245

[ref133] Du J.; Zhou Y.; Su X.; Yu J. J.; Khan S.; Jiang H.; Kim J.; Woo J.; Kim J. H.; Choi B. H.; et al. Sirt5 is a NAD-dependent protein lysine demalonylase and desuccinylase. Science 2011, 334 (6057), 806–809. 10.1126/science.1207861. 22076378 PMC3217313

[ref134] Li L.; Shi L.; Yang S.; Yan R.; Zhang D.; Yang J.; He L.; Li W.; Yi X.; Sun L.; et al. SIRT7 is a histone desuccinylase that functionally links to chromatin compaction and genome stability. Nat. Commun. 2016, 7, 12235 10.1038/ncomms12235. 27436229 PMC4961794

[ref135] Zhang X.; Cheng K.; Ning Z.; Mayne J.; Walker K.; Chi H.; Farnsworth C. L.; Lee K.; Figeys D. Exploring the microbiome-wide lysine acetylation, succinylation, and propionylation in human gut microbiota. Anal. Chem. 2021, 93 (17), 6594–6598. 10.1021/acs.analchem.1c00962. 33885279

[ref136] Zhang X.; Ning Z.; Mayne J.; Yang Y.; Deeke S. A.; Walker K.; Farnsworth C. L.; Stokes M. P.; Couture J.-F.; Mack D.; et al. Widespread protein lysine acetylation in gut microbiome and its alterations in patients with Crohn’s disease. Nat. Commun. 2020, 11 (1), 4120 10.1038/s41467-020-17916-9. 32807798 PMC7431864

